# Biodegradable-Polymer or Durable-Polymer Stents in Patients at High Bleeding Risk: A Randomized, Open-Label Clinical Trial

**DOI:** 10.1161/CIRCULATIONAHA.123.065448

**Published:** 2023-08-25

**Authors:** Marco Valgimigli, Adrian Wlodarczak, Ralph Tölg, Béla Merkely, Henning Kelbæk, Jacek Legutko, Stefano Galli, Matthieu Godin, Gabor G. Toth, Thibault Lhermusier, Benjamin Honton, Peter Laurenz Dietrich, Francis Stammen, Bert Ferdinande, Johanne Silvain, Davide Capodanno, Guillaume Cayla

**Affiliations:** 1 Cardiocentro Ticino Institute, Ente Ospedaliero Cantonale (EOC), Università della Svizzera Italiana, Lugano, Switzerland (M.V.).; 2 Poland Miedziowe Centrum Zdrowia Lubin, Poland (A.W.).; 3 Herzzentrum der Segeberger Kliniken GmbH, Bad Segeberg, Germany (R.T.).; 4 Semmelweis University, Heart and Vascular Center, Budapest, Hungary (B.M.).; 5 Department of Cardiology, Zealand University Hospital, Roskilde, Denmark (H.K.).; 6 Department of Interventional Cardiology, Institute of Cardiology, Jagiellonian University Medical College, John Paul II Hospital, Krakow, Poland (J.L.).; 7 Department of Interventional Cardiology, Centro Cardiologico Monzino, Istituto di Ricovero e Cura a Carattere Scientifico, Milan, Italy (S.G.).; 8 Department of Cardiology, Clinique Saint Hilaire, Rouen, France (M.G.).; 9 University Heart Center Graz, Department of Cardiology, Medical University Graz, Austria (G.G.T.).; 10 Hôpital de Rangueil, Fédération de Cardiologie, Pôle Cardio-vasculaire et Métabolique, Toulouse, France (T.L.).; 11 Department of Interventional Cardiology, Clinique Pasteur, Toulouse, France (B.H.).; 12 Division of Cardiology, Stadtspital Triemli, Zurich, Switzerland (P.L.D.).; 13 Department of Cardiology, AZ Delta, Roeselare, Belgium (F.S.).; 14 Department of Cardiology, Hospital Oost-Limburg Genk, Belgium (B.F.).; 15 Sorbonne Université, ACTION Group, INSERM UMRS 1166, Hôpital Pitié-Salpêtrière (AP-HP), Institut de Cardiologie, Paris, France (J.S.).; 16 Division of Cardiology, Azienda Ospedaliero Universitaria Policlinico “G. Rodolico-San Marco,” University of Catania, Italy (D.C.).; 17 Cardiology Department, Nîmes University Hospital, Montpellier University, Nîmes, France (G.C.).

**Keywords:** aspirin, drug-eluting stents, hemorrhage, risk, therapeutics

## Abstract

**BACKGROUND:**

Limited information is available on the comparative efficacy and safety of different stent platforms in patients at high bleeding risk undergoing an abbreviated dual antiplatelet therapy duration after percutaneous coronary intervention (PCI). The aim of this study was to compare the safety and effectiveness of the biodegradable-polymer sirolimus-eluting stent with the durable-polymer zotarolimus-eluting stent in patients at high bleeding risk receiving 1 month of dual antiplatelet therapy after PCI.

**METHODS:**

The Bioflow-DAPT Study is an international, randomized, open-label trial conducted at 52 interventional cardiology hospitals in 18 countries from February 24, 2020, through September 20, 2021. Patients with a clinical indication to PCI because of acute or chronic coronary syndrome who fulfilled 1 or more criteria for high bleeding risk were eligible for enrollment. Patients were randomized to receive either biodegradable-polymer sirolimus-eluting stents or durable-polymer, slow-release zotarolimus-eluting stents after successful lesion preparation, followed by 1 month of dual antiplatelet therapy and thereafter single antiplatelet therapy. The primary outcome was the composite of death from cardiac causes, myocardial infarction, or stent thrombosis at 1 year, and was powered for noninferiority, with an absolute margin of 4.1% at 1-sided 5% alpha.

**RESULTS:**

A total of 1948 patients at high bleeding risk were randomly assigned (1:1) to receive biodegradable-polymer sirolimus-eluting stents (969 patients) or durable-polymer zotarolimus-eluting stents (979 patients). At 1 year, the primary outcome was observed in 33 of 969 patients (3.6%) in the biodegradable-polymer sirolimus-eluting stent group and in 32 of 979 patients (3.4%) in the durable-polymer zotarolimus-eluting stent group (risk difference, 0.2 percentage points; upper boundary of the 1-sided 95% CI, 1.8; upper boundary of the 1-sided 97.5% CI, 2.1; *P*<0.0001 for noninferiority for both tests).

**CONCLUSIONS:**

Among patients at high risk for bleeding who received 1 month of dual antiplatelet therapy after PCI, the use of biodegradable-polymer sirolimus-eluting stents was noninferior to the use of durable-polymer zotarolimus-eluting stents with regard to the composite of death from cardiac causes, myocardial infarction, or stent thrombosis.

**REGISTRATION:**

URL: https://www.clinicaltrials.gov; Unique identifier: NCT04137510.

**Clinical Perspective**

**What Is New?**

- The 1-year primary composite outcome of death from cardiac causes, myocardial infarction, or stent thrombosis with the biodegradable-polymer sirolimus-eluting stent was noninferior to the durable-polymer zotarolimus-eluting stent, according to predefined noninferiority criteria.

**What Are the Clinical Implications?**

- In patients at high bleeding risk who are meant to receive 1 month of dual antiplatelet therapy after percutaneous coronary intervention, the use of biodegradable-polymer sirolimus-eluting stents is a reasonable alternative to the use of durable-polymer zotarolimus-eluting stents at the time of intervention.

More than one-third of patients treated with percutaneous coronary intervention (PCI) are considered to be at high bleeding risk and are frequently excluded from stent trials.^1,2^ Randomized and nonrandomized studies have shown that an abbreviated dual antiplatelet regimen of 1 month after PCI reduces bleeding without compromising safety compared with more prolonged treatment durations in patients at high bleeding risk.^3,4^ However, there is limited information on the comparative efficacy and safety of different stent platforms in patients at high bleeding risk undergoing an abbreviated dual antiplatelet therapy (DAPT) duration after PCI.

Previous randomized controlled studies have shown better outcomes with drug-eluting stents than bare-metal stents in patients at high bleeding risk who were treated with 1 or 6 months of DAPT after PCI.^3,5–7^ Onyx ONE (A Randomized Controlled Trial With Resolute Onyx in One Month Dual Antiplatelet Therapy for High-Bleeding Risk Patients) is the only study that compared 2 contemporary drug-eluting stent platforms in patients at high bleeding risk undergoing 1 month of DAPT and showed that durable-polymer zotarolimus-coated stents were associated with noninferior outcomes to polymer-free umirolimus-coated stents.^8^ No study has compared a biodegradable-polymer stent platform with durable-polymer stents in such patients.

We conducted a randomized trial to compare the safety and effectiveness of the biodegradable-polymer sirolimus-eluting stent with the durable-polymer zotarolimus-eluting stent in patients at high bleeding risk receiving 1 month of DAPT after PCI.

## METHODS

### Data Availability

The data that support the findings of this study are available from the corresponding author upon reasonable request.

### Trial Oversight

The Bioflow-DAPT Study (URL: https://www.clinicaltrials.gov; Unique identifier: NCT04137510) was a randomized, multicenter, open-label, noninferiority trial funded by Biotronik, designed jointly by the executive committee and the sponsor, and approved by the institutional review board at each center (as described in the Supplemental Material). The methods have been published elsewhere.^9^ All the statistical analyses were performed by the sponsor and validated by an independent statistician. The first draft of the manuscript was written by the first author. All the authors had full access to the data, revised the manuscript, supported the decision to submit the manuscript for publication, and vouch for the accuracy and completeness of the data and for the fidelity of the trial to the protocol. Requests from qualified investigators for data from the trial will be considered by its executive steering committee.

### Patients

Patients with a clinical indication to PCI because of an acute or chronic coronary syndrome were considered candidates to participate in the trial if they fulfilled 1 or more criteria for high bleeding risk (as described in the Supplemental Material), which closely resemble the criteria proposed by the Academic Research Consortium,^1,2^ or had a PRECISE-DAPT score (Predicting Bleeding Complications in Patients Undergoing Stent Implantation and Subsequent Dual Antiplatelet Therapy) of at least 25 points^10^ and were eligible for 1 month of DAPT after the procedure. A clinically indicated planned staged procedure was not allowed. A full list of inclusion and exclusion criteria is presented in the Supplemental Material. The study was approved by an institutional review committee, and all patients gave written informed consent.

### Trial Procedures

Patients were randomly assigned in a 1:1 ratio to receive open-label the biodegradable-polymer sirolimus-eluting stent (Orsiro Mission; Biotronik) or the durable-polymer, slow-release zotarolimus-eluting stent (Resolute Onyx; Medtronic) after successful lesion preparation. Randomization was concealed using a web-based system; randomization sequences were computer-generated, blocked with randomly selected block sizes of 2 or 4, and were stratified by country, site, diabetes status, and intended use of a single antiplatelet drug (aspirin or a P2Y_12_ inhibitor) after 30 days of DAPT. The use of antithrombotic drugs during the index procedure was according to guidelines and standard of care, including a loading dose of a P2Y_12_ inhibitor or aspirin for participants who did not receive the medications for at least 7 days previously. After the procedure, patients were given a prescription for 1 month of DAPT that included a daily dose (75 to 100 mg) of aspirin and a P2Y_12_ inhibitor. At the 1-month follow-up visit, eligibility for discontinuation of DAPT was reassessed by investigators. Patients who were adherent to treatment and free from recurrent events were eligible for treatment discontinuation and received a prescription for either aspirin or a P2Y_12_ monotherapy at the discretion of the treating physician. Participants who were ineligible for treatment discontinuation were not excluded from the study and were followed through for 12 months.

Patients eligible for follow-up visits were those who received at least 1 study stent. During follow-up visits, which occurred preferably on-site, at 30 (range, −2 to 7), 180 (±30), and 365 (±30) days after randomization, ischemic status, quality-of-life questionnaires, self-reported adherence to antiplatelet medications, and any adverse event data were collected.

Complete source data verification was implemented in 541 patients (28.2%), including the first 2 patients enrolled at each site and a randomly selected cohort of 25% of the remaining patients per site.

### Trial Outcomes

All outcomes were adjudicated by a committee of members who were unaware of trial-group assignments. The primary outcome was the composite of death from cardiac causes, myocardial infarction (MI), or definite or probable stent thrombosis at 1 year. Secondary outcomes included the components of the primary outcome, major adverse cardiac and cerebrovascular events (defined as death, MI, or stroke), major adverse cardiac events (defined as death, MI, or target-vessel revascularization), stroke, target-vessel or lesion failure, and bleeding events. MI was primarily defined according to the Academic Research Consortium–2 definition.^11^ Other MI definitions were prespecified as secondary outcomes.^12–14^ A detailed list of all outcomes and their definitions (MI,^13–15^ bleeding,^16–18^ angina,^19–21^ cardiogenic shock,^22^ cardiac or vascular death,^23^ and heart failure^24^) is provided in the Expanded Methods in the Supplemental Material and Tables S1 through S5.

### Statistical Analysis

On the basis of the LEADERS FREE (A Randomized Clinical Evaluation of the BioFreedom Stent) results,^25^ we assumed that 9.4% of the patients in each group would have a primary outcome event at 1 year. A noninferiority margin of 4.1 percentage points was chosen based on the Onyx ONE trial.^8^ We calculated that a sample of 876 patients in each group would provide 90% power to show noninferiority on the basis of the Farrington–Manning test^26^ at a 1-sided type 1 error of 0.05. To compensate for a 10% attrition rate, 1948 patients were randomized. The results for the primary outcome were derived from the intention-to-treat population.

Additional analyses were performed in the per-protocol and as-treated populations; definitions of the analysis populations are provided in the Expanded Methods in the Supplemental Material. The 95% CIs presented in this article have not been adjusted for multiple comparisons; therefore, inferences drawn from these intervals may not be reproducible. In a post hoc analysis, the upper boundary of the 1-sided 97.5% CI, which corresponds to a 1-sided type 1 error of 0.025 for the primary analysis results, was also calculated.

Categorical data are reported as percentages and were compared using the Fisher exact test. Continuous data are reported as means with SDs and were tested with the use of 2-sample Student *t* tests; rates are based on all the patients who underwent randomization and had data that could be evaluated. For prespecified subgroup analyses, the interaction term between treatment groups and subgroups was evaluated with the use of logistic regression. Cumulative-incidence curves with Kaplan-Meier estimates were generated. Post hoc landmark analyses were performed with the use of cut-offs at 30 days, which corresponded to the planned date of the discontinuation of DAPT. All statistical analyses were performed with SAS software, version 9.4.

## RESULTS

### Patients

Of 2769 patients enrolled in the trial, 1948 (at 52 sites in 18 countries) underwent randomization between February 24, 2020, and September 20, 2021; a total of 969 patients were assigned to and 952 received at least 1 biodegradable-polymer sirolimus-eluting stent, whereas 979 were assigned to and 969 received at least 1 durable-polymer zotarolimus-eluting stent (Figure S1). A total of 18 patients (1.9%) assigned to the biodegradable-polymer sirolimus-eluting stent group and 23 (2.3%) patients assigned to the durable-polymer zotarolimus-eluting stent group were lost to follow-up or withdrew consent. The per-protocol population comprised 892 patients in the biodegradable-polymer sirolimus-eluting stent group and 894 patients in the durable-polymer zotarolimus-eluting stent group (Figure S1).

Baseline characteristics and clinical presentation of the trial groups are shown in Table 1. The mean age was 75.8±8.4 years; 68.6% were men, 31.4% had diabetes, 33.1% had chronic kidney disease, 21.4% had heart failure, and 13.2% had a previous cerebrovascular event. Of 1948 patients included, 573 (29.4%) underwent coronary intervention for acute coronary syndrome. The mean number of high bleeding risk criteria per patient was 1.93±1.13, and 1040 patients (53.4%) met 2 or more criteria (Table 2). The most common high–bleeding risk qualifying features were 75 years of age or older and oral anticoagulation use.

**Table 1.**
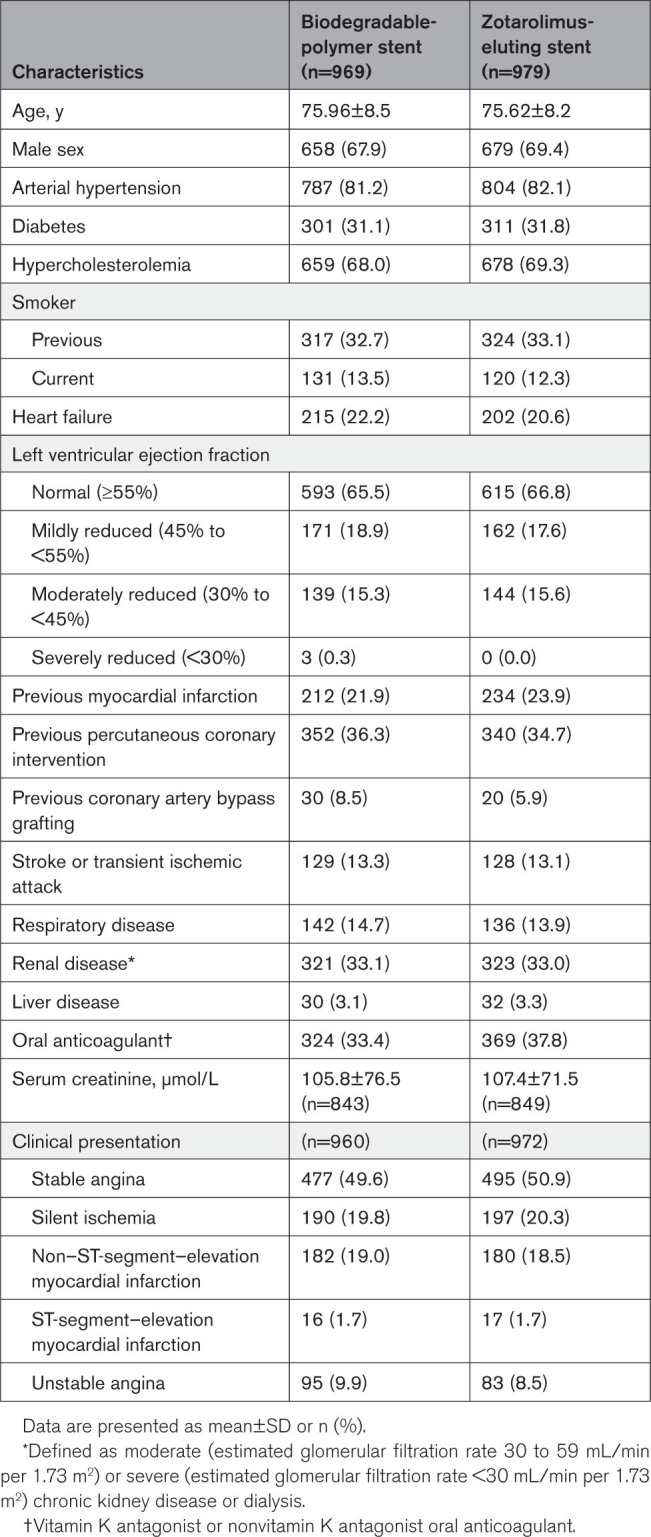
Baseline Characteristics and Clinical Presentation

**Table 2.**
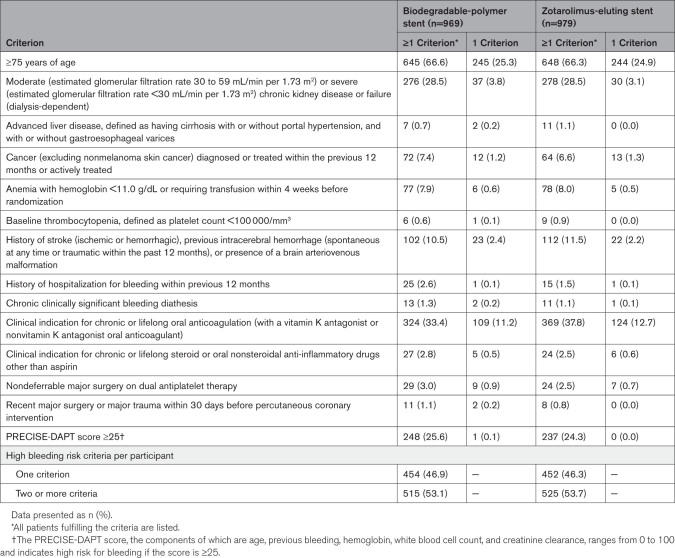
Criteria for High Bleeding Risk

### Procedural Characteristics

Vascular access was radial in >80% of the patients, and the left anterior descending artery was the most frequently treated vessel. Most patients had complex lesions (Tables S6 through S8). Moderate to severe calcification was present in more than one-third of patients, and a bifurcation lesion was treated in >30% of the patients. Most patients underwent a single-vessel intervention, and the mean total stent length was 37 mm. No patient assigned to the biodegradable-polymer sirolimus-eluting stent crossed over to receive the zotarolimus-eluting stent; 3 patients assigned to the zotarolimus-eluting stent crossed over to receive the biodegradable-polymer sirolimus-eluting stent. Device success occurred in 1305 of 1350 lesions (96.7%) in patients in the biodegradable-polymer sirolimus-eluting stent and in 1304 of 1336 lesions (97.6%) in patients in the zotarolimus-eluting stent group.

The timing of the discontinuation of DAPT was similar in the 2 groups. At 30 days, DAPT had been prescribed in 769 of 934 patients (82.3%) in the biodegradable-polymer sirolimus-eluting stent group and in 807 of 961 patients (84.0%) in the zotarolimus-eluting stent group (Table S9). At 6 months, 42 of 912 patients (4.6%) in the biodegradable-polymer sirolimus-eluting stent group and 36 of 928 patients (3.9%) in the zotarolimus-eluting stent group were on DAPT; at 1 year, 24 of 594 patients (4.0%) and 22 of 604 patients (3.6%), respectively, were taking DAPT. Single antiplatelet agent therapy at 1 year consisted of aspirin for 247 of 594 patients (41.6%) in the biodegradable-polymer sirolimus-eluting stent and for 253 of 604 patients (41.9%) in the zotarolimus-eluting stent group, and consisted of a P2Y_12_ inhibitor for 194 patients (32.6%) and 204 patients (33.8%), respectively (Tables S9 and S10). Adherence to antiplatelet therapy is shown in Figure S2.

### Primary Outcome

**Figure 1.**
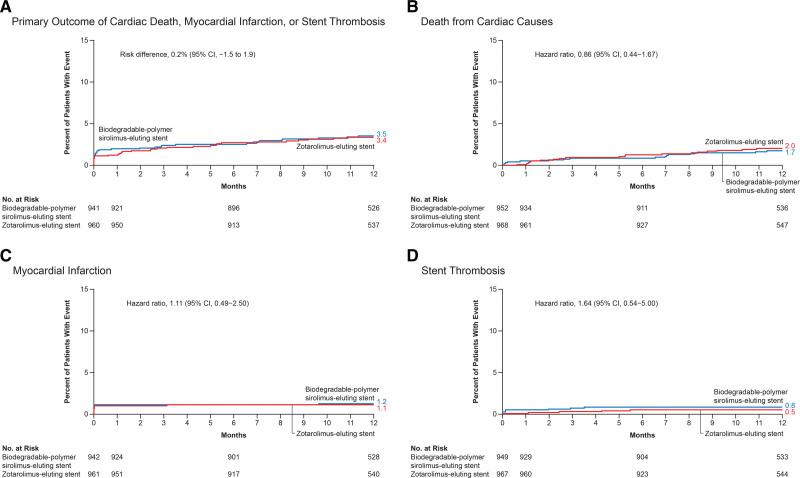
**Kaplan-Meier time-to-event curves for the primary outcome and its components.** The primary outcome was the composite of death from cardiac causes, myocardial infarction, and definite or probable stent thrombosis, according to the Academic Research Consortium–2 definition. Data for patients who were lost to follow-up or withdrew from the trial before 1 year were censored at the end of follow-up.

At 1 year, the primary outcome (a composite of death from cardiac causes, MI, or stent thrombosis) had occurred in 33 of 969 patients (3.6%) in the biodegradable-polymer sirolimus-eluting stent group and in 32 of 979 patients (3.4%) in the zotarolimus-eluting stent group (risk difference, 0.2 percentage points; upper boundary of the 1-sided 95% CI, 1.8; *P*<0.0001; upper boundary of the 1-sided 97.5% CI, 2.1; *P*<0.0001 for noninferiority; Table 3). Cumulative-incidence curves for the primary outcome and its components are shown in Figure 1. Noninferiority was confirmed in per-protocol analyses at the 1-sided level of 0.05 and at the 1-sided level of 0.025 (risk difference, 0.4 percentage points; upper boundary of the 1-sided 95% CI, 2.0; *P*<0.0001; upper boundary of the 1-sided 97.5% CI, 2.3; *P*<0.0001 for noninferiority).

**Table 3.**
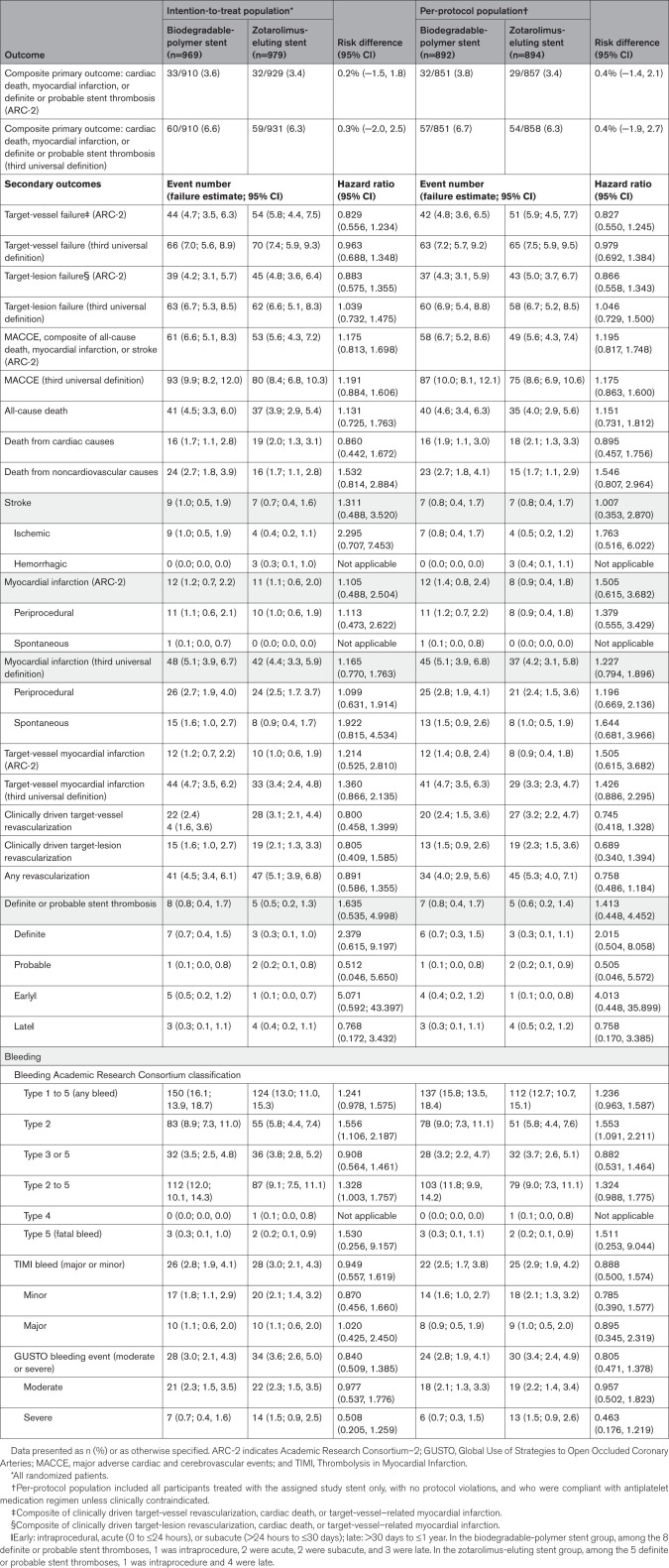
**Composite Primary Outcome and Secondary Outcomes (Intention-to-Treat and Per-Protocol Populations**)

### Secondary Outcomes

**Figure 2.**
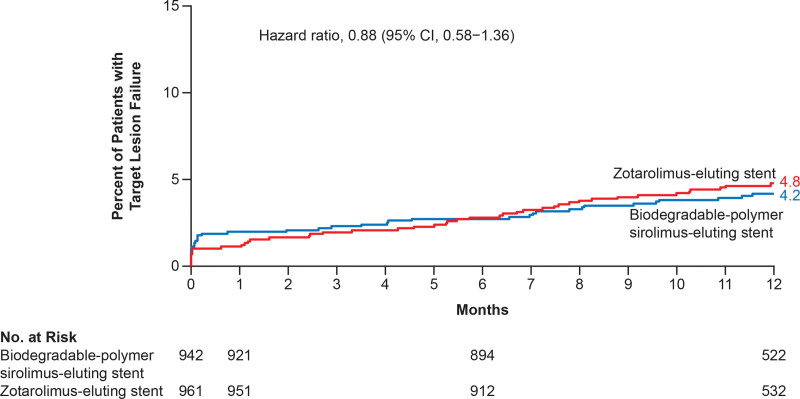
**Kaplan-Meier time-to-event curves for target-lesion failure.** Target-lesion failure was defined as a composite of death from cardiac causes, target-vessel myocardial infarction, or clinically indicated target-lesion revascularization. Data for patients who were lost to follow-up or withdrew from the trial before 1 year were censored at the end of follow-up.

At 1 year, target-lesion failure had occurred in 39 patients (4.2%) in the biodegradable-polymer sirolimus-eluting stent group and in 45 patients (4.8%) in the zotarolimus-eluting stent group (Figure 2; Table 3).

The incidence of all-cause death (4.5% and 3.9%, respectively), MI (1.2% and 1.1%), definite or probable stent thrombosis (0.8% and 0.5%), stroke (1.0% and 0.7%), and clinically driven target-vessel (2.4% and 3.1%) or lesion (1.6% and 2.1%) revascularization did not differ with biodegradable-polymer sirolimus-eluting stents or zotarolimus-eluting stents (Table 3).

Any bleeding events and bleeding that met the Bleeding Academic Research Consortium type 3 through 5 criteria occurred in 150 (16.1%) and 32 (3.5%) patients in the biodegradable-polymer sirolimus-eluting stent group and in 124 (13.0%) and 36 (3.8%) patients in the zotarolimus-eluting stent group, respectively (Table 3).

### Additional Analyses

The effects of biodegradable-polymer sirolimus-eluting stent or zotarolimus-eluting stent on the incidence of the primary outcome were consistent across prespecified subgroups (Figure S3).

When the third universal MI definition was implemented in a prespecified analysis of the primary outcome, the event rates were 6.6% in the biodegradable-polymer sirolimus-eluting stent group and 6.3% in the zotarolimus-eluting stent group (risk difference, 0.3 percentage points; upper boundary of the 1-sided 95% CI, 2.2; upper boundary of the 1-sided 97.5% CI, 2.6; Figure S4; Table 3).

Time-to-event curves with landmark analysis at 30 days analyzed according to the Kaplan-Meier method are shown in Figures S4 through S8. Between 0 and 30 days, the primary outcome occurred in 2.0% of patients in the biodegradable-polymer sirolimus-eluting stent group and in 1.2% of those in the zotarolimus-eluting stent group. Between 31 days and 1 year, the comparable percentages were 1.6% and 2.1%, respectively. Exploratory analyses on event rates for patients who underwent 100% monitoring versus those for whom risk-based monitoring was implemented showed no systematic underreporting in the former group (Table S11).

## DISCUSSION

In the Bioflow-DAPT Study, we found that for patients at high risk for bleeding and received 1 month of DAPT after PCI, the biodegradable-polymer sirolimus-eluting stent was noninferior to the durable-polymer zotarolimus-eluting stent at 1 year with respect to the primary outcome of death from cardiac causes, MI, or stent thrombosis.

This is the first study to show that clinical outcomes after biodegradable-polymer sirolimus-eluting stents did not differ compared with durable-polymer zotarolimus-eluting stents in patients at high risk for bleeding undergoing 1 month of DAPT after intervention. The current study builds on previous evidence.^3^ In a single previous trial, net and major adverse events were noninferior, and major or clinically relevant nonmajor bleeding was lower with 1 month compared with at least 3 months of DAPT in patients at high bleeding risk after biodegradable-polymer sirolimus-eluting stent implantation.^3^ No study has compared different durations of DAPT after durable-polymer drug-eluting stents in patients at high risk for bleeding, whereas the durable-polymer zotarolimus-eluting stent was found to be noninferior to polymer-free drug-coated stents in patients at high bleeding risk undergoing 1 month of DAPT after PCI.^8^ This study found that the incidences of primary and secondary outcomes at 1 year did not differ between durable-polymer zotarolimus-eluting and polymer-free drug-coated stents after protocol-mandated cessation of DAPT at landmark analysis at 30 days, with the exception of a lower rate of MI with durable-polymer zotarolimus-eluting stent.^8^ In the current study, primary and secondary outcomes at 1 year did not differ for patients with biodegradable-polymer sirolimus-eluting or durable-polymer zotarolimus-eluting stents at landmark analysis at 30 days, although the rate of target-vessel failure was lower with biodegradable-polymer sirolimus-eluting stents between 31 days and 1 year. In a recent network meta-analysis of 77 trials involving 99 039 patients, the biodegradable-polymer sirolimus-eluting stent was associated with a lower target-lesion failure rate compared with the durable-polymer everolimus or zotarolimus-eluting stents.^27^ The 5-year follow-up of BIOFLOW-V (Safety and Effectiveness of the Orsiro Sirolimus Eluting Coronary Stent System in Subjects With Coronary Artery Lesions) also suggested lower rates of target-vessel MI and stent thrombosis with the biodegradable-polymer sirolimus-eluting stent compared with the durable-polymer everolimus-eluting stent.^28^ However, in previous studies, duration of DAPT was not protocol-mandated at 30 days, and in none were patients at high bleeding risk exclusively included.

The current study had similar inclusion and exclusion criteria compared with previous investigations assessing different stents in patients at high risk for bleeding.^29^ On the basis of previous studies and mainly informed by the most recent ones,^8^ we expected that slightly fewer than 1 in 10 patients would incur a primary outcome event in both stent groups and selected a noninferiority margin preserving an acceptable fraction of the expected treatment effect. The observed frequency of primary outcome events was nearly 3-fold lower than expected. Unlike previous trials, we selected the Academic Research Consortium–2 MI definition in the primary outcome. A prespecified analysis of the primary outcome based on the third universal definition of MI resulted in doubled event rates in the 2 study groups. Periprocedural MI occurred in 7.8% of patients in the polymer-free coated stent group and in 9.3% of patients in the zotarolimus-eluting stent group in the Onyx ONE trial,^8^ and were ascertained mostly by angiographic evidence of ischemia at core laboratory analysis. In our study, angiograms of the interventions were not independently assessed in a core laboratory; periprocedural MI, according to the third universal definition, occurred in 2.7% of patients with biodegradable-polymer sirolimus-eluting stents and 2.4% with durable-polymer zotarolimus-eluting stents. The proportion of patients with an acute MI at presentation was lower than in previous trials.^6,8,25^ This may further explain the low rates of cardiovascular death and stent thrombosis in our trial versus previous studies. The protocol mandated the ascertainment of left ventricular ejection fraction before inclusion, which limited the participation of patients with ST-segment–elevation MI and may have limited the inclusion of patients with acute coronary syndromes, in general. The frequency of actionable or major bleeding was comparable in our study compared with previous trials.^3,6–8,25^

The most important limitation of the trial is that the incidences of outcome events at 1 year were lower than expected on the basis of data from the Onyx ONE trial.^8^ As a result, the noninferiority margin was large relative to the incidence of major adverse cardiac events. Thus, noninferiority of the biodegradable-polymer sirolimus-eluting stent would have been shown even if the incidence was up to 75% higher than in the durable-polymer zotarolimus-eluting stent group. However, the actual incidences in the 2 groups did not differ. The prespecified secondary analysis with use of the third universal definition of MI, instead of the Academic Research Consortium–2 MI definition in the primary outcome, was associated with incidences of outcome events at 1 year that were nearly twice as high, which did not differ in the 2 stent groups. Other limitations of our trial should also be considered. Treatments were open-label, and the decision to continue aspirin or a P2Y_12_ inhibitor after 30 days of DAPT was at the discretion of physicians. Complete source data verification was implemented in a random cohort of 28.2% of the patients. Exploratory analyses suggested similar rates of adverse events in patients with versus those without complete monitoring. The angiograms of the index procedure were not assessed by an independent core laboratory. Sample size calculation for noninferiority testing was based on 1-sided 5% alpha, as in previous trials.^30^ Post hoc analysis based on 2-sided 5% alpha testing provided consistent results.

### Conclusions

For patients at high risk for bleeding, a strategy of PCI with biodegradable-polymer sirolimus-eluting stents followed by 30 days of DAPT was noninferior to durable-polymer zotarolimus-eluting stents with respect to the incidence of death from cardiac causes, MI, or stent thrombosis.

## ARTICLE INFORMATION

### Acknowledgments

Editorial support was provided by Sophie Rushton-Smith, PhD (MedLink Healthcare Communications), funded by Biotronik.

### Sources of Funding

The Bioflow-DAPT trial was conducted with support from Biotronik. The sponsor was responsible for site selection, data monitoring, and overall clinical trial management. All statistical analyses were performed by the sponsor and validated by an independent statistician.

### Disclosures

Dr Valgimigli reports grants and personal fees from Terumo and personal fees from AstraZeneca, Alvimedica/CID, Abbott Vascular, Daiichi Sankyo, Bayer, CoreFLOW, IDORSIA Pharmaceuticals Ltd, Universität Basel, Department Klinische Forschung, Vifor, Bristol Myers Squibb SA, Biotronik, Boston Scientific, Medtronic, Vesalio, Novartis, Chiesi, and PhaseBio. Dr Wlodarczak has no conflicts of interest to report. Dr Tölg reports personal fees for lectures from Biotronik. Dr Merkely reports personal fees from Abbott, AstraZeneca, Biotronik, Boehringer Ingelheim, CSL Behring, Daiichi Sankyo, Duke Clinical Research Institute, Medtronic, and Novartis, and institutional grants from Abbott, AstraZeneca, Biotronik, Boehringer Ingelheim, Boston Scientific, Bristol Myers Squibb, CSL Behring, Daiichi Sankyo, Duke Clinical Institute, Eli Lilly, Medtronic, Novartis, Terumo, and VIFOR Pharma. Dr Kelbæk has no conflicts of interest to report. Dr Legutko reports lecture fees from Abiomed, AstraZeneca, Berlin Chemie-Menarini, Abbott, Bayer AG, Ely Lilly, Medtronic, MSD, Philips-Volcano, Procardia, Sanofi, and Novartis; meeting or travel fees from Abbott, Boston, Medtronic, and Abiomed; and data safety monitoring board or advisory board fees from AstraZeneca, Berlin Chemie-Menarini, Abbott, Sanofi, Gedeon Richte, Hemolens Diagnostics, Novartis, and Ferrer. Dr Galli reports consulting fees from BIOACADEMY and lecture fees from PCR. Dr Godin reports lecture fees from B. Braun, Medtronic, and Edwards, and data safety monitoring board or advisory board fees from Terumo and Microport. Dr Toth reports grants and personal fees from Terumo, Abbott Vascular, Biotronik, and Medtronic. Dr Lhermusier reports consulting fees from Abbott and meeting or travel fees from Medtronic. Dr Honton reports consulting fees from Medtronic, Shockwave, Terumo, and Microport; lecture fees from Shockwave, Medtronic, and Boston; and data safety monitoring board or advisory board fees from Microport, Terumo, and Medtronic. Dr Dietrich reports educational event fees to his institution from Cordis. Drs. Stammen and Ferdinande have no conflicts of interest to report. Dr Silvain reports consulting fees from AstraZeneca, Bayer Health Care SAS, CSL Behring SA, Sanofi-Aventis France, and Zoll, and stock options without payment at 4P-Pharma. Dr Capodanno reports honoraria fees from Biotronik and honoraria fees to his institution from Medtronic. Dr Cayla reports personal consulting fees from Edwards, Medtronic, and Microport CRM, and personal honoraria fees from Amgen, AstraZeneca, Abbott, Bayer, Biotronik, Bristol-Myers Squibb, Edwards, Microport, Medtronic, Pfizer, and Sanofi-Aventis.

### Supplemental Material

Bioflow-DAPT Trial Committees and Investigators

Expanded Methods

Figures S1–S8

Tables S1–S11

Appendix

## Supplementary Material
