# Supplementary material for: Biodegradable-Polymer or Durable-Polymer Stents in Patients at High Bleeding Risk: A Randomized, Open-Label Clinical Trial
Source: Circulation. 2023 Aug 25;148(13):989–99. doi: 10.1161/CIRCULATIONAHA.123.065448 (PMC10516164; doi:10.1161/CIRCULATIONAHA.123.065448)
Supplement: Supplementary file 1 [file cir-148-0989-s001.pdf]

SUPPLEMENTAL MATERIAL

Biodegradable-Polymer or Durable-Polymer Stents in Patients at High Bleeding Risk. A Randomized, Open-Label Clinical Trial

Marco Valgimigli, et al.

Contents

EXPANDED METHODS 2
Antiplatelet Regimens 2
Antiplatelet Treatment Adherence 2
Study criteria 3
Analysis Sets 3
Intention-to-treat (ITT) Set 3
As Treated Set 3
Per-Protocol (PP) Set 4
Outcome Definitions 5
Bleeding Classifications 5
Cardiac Death 6
Cardiogenic Shock[22] 6
Clinically Driven Target Lesion Revascularization 6
Clinically Driven Target Vessel Revascularization 6
Death[23] 6
Major Adverse Cardiac Events 7
Major Adverse Cardiac And Cerebrovascular Events (MACCE) 7
Myocardial Infarction 7
Target Lesion Failure 11
Target Lesion Revascularization 11
Target Vessel Failure 11
Target Vessel Myocardial Infarction 11
Target Vessel Revascularization 11
Table S1. Braunwald Classification[19] 12
Table S2. Canadian Cardiovascular Society Angina Classification[20,21] 13
Table S3. Third Universal Definition of Myocardial Infarction[15] 14
Table S4. Fourth Universal Definition of Myocardial Infarction[12] 15
Table S5. Stent Thrombosis – Academic Research Consortium-2 Definition[11] 17
Table S6. Procedural Characteristics at the Patient Level[a] 18
Table S7. Procedural Characteristics at the Lesion Level[a] 19
Table S8. Characteristics of Target Lesions[a] 20
Table S9. Medications 21
Table S10. Additional Information on Type of Antiplatelet Therapy After Randomization 23
Table S11. Primary and Secondary Outcomes in the Intention-To-Treat Population of Patients with Full Source Data Verification Versus Patients without Full source Data Verification 25
Figure S1. Flow Diagram 27
Figure S2. Adherence to Antiplatelet Therapy 28
Figure S3. Incidence of the Composite Primary Outcome (Academic Research Consortium-2 Definition) in Prespecified Subgroups 29
Figure S4. Cumulative Incidence of the Composite Primary Outcome (Third Universal Definition) 30
Figure S5. Cumulative Incidence of the Composite Primary Outcome (Academic Research Consortium-2 Definition) in a Landmark Analysis 31
Figure S6. Cumulative Incidence of (A) Death from Cardiac Causes; (B) Myocardial Infarction (Academic Research Consortium-2 Definition); and (C) Definite or Probable Stent Thrombosis in a Landmark Analysis 32
Figure S7. Cumulative Incidence of Target Lesion Failure in a Landmark Analysis 34
Figure S8. Cumulative Incidence of Target Vessel Failure in a Landmark Analysis 35
BIOFLOW-DAPT Trial: Committees and Investigators 36
Countries, Investigators, and Numbers of Patients Enrolled 37

EXPANDED METHODS

Antiplatelet Regimens

No concomitant indication for oral anticoagulation

In eligible patients without indication for oral anticoagulation, dual antiplatelet therapy is discontinued at 1 month after randomization, followed by single antiplatelet therapy with either aspirin or $P2Y_{12}$ monotherapy (at investigator's discretion) for the duration of the study.

Concomitant indication for oral anticoagulation

Patients with concomitant indication for oral anticoagulation receive triple therapy (dual antiplatelet therapy + vitamin K antagonist) or a direct oral anticoagulant) for 30 days following the index procedure. At 1 month, these subjects discontinue dual antiplatelet therapy and receive single antiplatelet therapy (aspirin or an oral $P2Y_{12}$ inhibitor) for a minimum of 5 months. Oral anticoagulation is continued until at least 12 months post-randomization. After study completion, antiplatelet or anticoagulant regimens followed current guidelines and standard of care. Selection of type and dosage of antiplatelet or anticoagulant options was in accordance with the corresponding authorization for use and locally approved regimens. Daily doses of allowed antiplatelet regimens included aspirin 75-162 mg once daily (od), clopidogrel 75 mg od, ticagrelor 90 mg twice daily, and prasugrel 10 mg or 5 mg od in patients weighing less than 60 kg or aged more than 75 years.

Daily doses of allowed direct oral anticoagulants included: apixaban 5 mg bid or apixaban 2.5 mg bid (if at least 2 among age ≥80 years, body weight ≤60 kg or serum creatinine level ≥1.5 mg/dL [or 133 mol/L]), dabigatran 150 mg bid or 110 mg bid, edoxaban 60 mg or 30 mg (if creatinine clearance was 30-50 mL/min or body weight ≤60 kg or there was concomitant use of verapamil, quinidine, or dronedarone) and rivaroxaban 20 mg or 15 mg od (if creatinine clearance 30-49 mL/min).

Finally, the dose intensity of vitamin K antagonist is monitored with a target international normalized ratio in the lower part of the recommended target range.

Antiplatelet Treatment Adherence

Adherence to single antiplatelet therapy was evaluated at 6-month and 12-month follow-up visits, as well as during any unscheduled visits. Subjects should have adhered to prescribed single antiplatelet therapy unless otherwise clinically indicated, tracking medication regimens in self-reported patient diaries. Any changes to antiplatelet or anticoagulant therapy were appraised by the investigator(s) and documented in the electronic case report form.

High bleeding risk criteria.

* PRECISE DAPT score is a 5-item bleeding risk score to predict the risk of out-of-hospital bleeding on dual antiplatelet therapy, which integrates prior bleeding, age, white blood cell count, creatinine clearance, and hemoglobin (web calculator at: http://www.precisedaptscore.com/predapt/webcalculator.html)

High bleeding risk criteria

Patients are at high bleeding risk if at least 1 of the following criteria applies:

a. Age of 75 years or more
b. Moderate (estimated glomerular filtration rate 30-59 mL/min/1.73 m^2) or severe (estimated glomerular filtration rate <30 mL/min/1.73 m^2) CKD or failure (dialysis dependent)
c. Advanced liver disease, defined as cirrhosis with or without portal hypertension and with or without gastroesophageal varices
d. Cancer (excluding nonmelanoma skin cancer) diagnosed or treated within the previous 12 months or actively treated
e. Anemia with hemoglobin <11.0 g/dL or requiring transfusion within 4 weeks prior to randomization
f. Baseline thrombocytopenia defined as a platelet count <100,000/mm^3
g. History of stroke (ischemic or hemorrhagic), previous intracerebral hemorrhage (spontaneous at any time or traumatic within the past 12 months) or presence of a brain arteriovenous malformation
h. Recent (≤12 months) hospitalization for bleeding
i. Chronic clinically significant bleeding diathesis
j. Clinical indication for chronic or lifelong oral anticoagulation

k. Clinical indication for chronic or lifelong steroid or oral nonsteroidal anti-inflammatory drug(s) (other than aspirin)
l. Nondeferrable major surgery on dual antiplatelet therapy
m. Recent major surgery or major trauma within 30 days before percutaneous coronary intervention
n. PRECISE DAPT score* ≥25

Study criteria
Inclusion criteria

Patients are eligible for study participation if all the following apply:

1. Acceptable candidate for treatment with a drug-eluting stent
2. At least one high bleeding risk criterion as defined in the box above
3. Age ≥18 years or the minimum age required for legal adult consent in the country of enrollment
4. Capable of providing written informed consent
5. Able to comply with all protocol and follow-up requirements, including agreement to discontinue dual antiplatelet therapy at 1 month
6. Eligible for treatment with dual antiplatelet therapy (aspirin plus a $P2Y_{12}$ inhibitor agent) for 1-month post index procedure

Exclusion criteria
Patients are not eligible if any of the following applies:

1. Previous stent or scaffold thrombosis in any coronary vessel
2. Known allergy to all types of $P2Y_{12}$ inhibitor (clopidogrel, ticagrelor, prasugrel, ticlopidine and cangrelor), aspirin, heparin, bivalirudin, L-605 cobalt-chromium alloy or one of its major elements (cobalt, chromium, tungsten, nickel), molybdenum, platinum and irridium, silicon carbide, PLLA, polymers, mTOR inhibiting drugs such as zotarolimus or sirolimus, or contrast media
3. Any target vessel revascularization within 9 months prior to the index procedure or previous before percutaneous coronary intervention of any nontarget vessel within 72 hours prior to or during the index procedure
4. Left ventricular ejection fraction <30% as evaluated by the most recent imaging exam (within 90 days pre/procedure or during the index procedure)
5. Unable for dual antiplatelet therapy discontinuation at 1 month, due to another condition requiring long-term dual antiplatelet therapy
6. Planned surgery or procedure necessitating $P2Y_{12}$ inhibitor and/or aspirin discontinuation within the first month post-index procedure
7. Active bleeding
8. Life expectancy less than 12 months
9. Participation in another trial
10. Pregnant and/or breastfeeding women
11. Unable to comply with the follow-up requirements
12. Need for an impartial witness to provide informed consent

Analysis Sets

Intention-to-treat (ITT) Set
The ITT set consists of all randomized subjects. Subjects will be analyzed "as-randomized", according to the group they were randomized to. In case that not more than 10% of patients received treatment different from what they were randomized to and not more than 10% had protocol violations, the main analysis of primary and secondary endpoints as well as informational endpoints will be performed on this population. The other analyses will be supportive.

As Treated Set
The "as treated" population includes subjects based on their actual treatment and is independent on their randomization group.

The Orsiro Mission group “As Treated” population will include subjects in the ITT population who were implanted only with the Orsiro Mission stent. Similarly, the Resolute Onyx “As Treated” group will include subjects in the ITT population who were implanted only with the Resolute Onyx Stent.

Per-Protocol (PP) Set
The PP subjects will include all subjects who were treated with the assigned study stent only, without any protocol violations that could significantly impact the completeness, accuracy and/or reliability of the trial data. The PP population will be identified during Report Review Meeting which will be performed before any analyses of study data.
Non-compliance to the following Inclusion Criteria will be considered protocol violations:

- Subject is acceptable candidate for treatment with DES
- Subject is considered at high bleeding risk (HBR), defined as meeting one or more of the following criteria at the time of enrollment:
    a. ≥ 75 years of age
    b. Moderate (estimated GFR 30-59 ml/min) or severe (estimated GFR < 30 ml/min) chronic kidney disease or failure (dialysis dependent)
    c. Advanced liver disease, defined as having cirrhosis with or without portal hypertension and with or without gastroesophageal varices.
    d. Cancer (excluding non-melanoma skin cancer) diagnosed or treated within the previous 12 months or actively treated
    e. Anemia with hemoglobin < 11.0 g/dL or requiring transfusion within 4 weeks prior to randomization
    f. Baseline thrombocytopenia defined as a platelet count <100,000/mm3
    g. History of stroke (ischemic or hemorrhagic), previous intracerebral hemorrhage (ICH) (spontaneous at any time or traumatic within the past 12 months) or presence of a brain arteriovenous malformation
    h. History of hospitalization for bleeding within the previous 12 months
    i. Chronic clinically significant bleeding diathesis
    j. Clinical indication for chronic or lifelong oral anticoagulation (OAC) (with a vitamin K antagonist or nonvitamin K OAC)
    k. Clinical indication for chronic or lifelong steroid or oral nonsteroidal anti-inflammatory drug(s) (NSAIDs), other than aspirin
    l. Nondeferrable major surgery on DAPT
    m. Recent major surgery or major trauma within 30 days before PCI
    n. PRECISE DAPT score ≥ 25
- Subject is ≥ 18 years or the minimum age required for legal adult consent in the country of enrollment
- Subject is capable (no legally authorized representative allowed) to provide written informed consent as approved by the Institutional Review Board (IRB)/Ethics Committee (EC) of the respective clinical site prior to any study related procedure
- Subject is eligible for dual antiplatelet therapy treatment with aspirin plus a P2Y12 inhibitor agent for 1-month post index procedure

Non-compliance to the following Exclusion Criteria will be considered protocol violations:

- Subject who previously experienced a stent or scaffold thrombosis in any coronary vessel
- Subject has a known allergy to all types of P2Y12 inhibitor (Clopidogrel, Ticagrelor, Prasugrel, Ticlopidine and Cangrelor; thus preventing the use of the appropriate P2Y12 inhibitor), aspirin, both heparin and bivalirudin, L-605 cobalt-chromium (Co-Cr) alloy or one of its major elements (cobalt, chromium, tungsten, nickel), molybdenum, platinum and irridium, silicon carbide, PLLA, polymers, mTOR inhibiting drugs such as zotarolimus or sirolimus, or contrast media
- Subject judged by physician as inappropriate for discontinuation from DAPT at 1 month following index procedure,due to another condition requiring chronic DAPT
- Subject with planned surgery or procedure necessitating discontinuation of P2Y12 inhibitor and/or aspirin within the first month post-index procedure
- Active bleeding at the time of inclusion
- Subject with a current medical condition with a life expectancy of less than 12 months
- Subject is pregnant and/or breastfeeding or intends to become pregnant during the duration of the study
- Subjects who need an impartial witness to give an informed consent

Subjects in PP population must be compliant with antiplatelet medication regimen unless clinically contraindicated. Compliance with antiplatelet medication regimen will be assessed based on self-reported adherence to medication. In case more than 10% of patients received treatment different from what they were randomized to or more than 10% had protocol violations, the main analysis of primary and secondary endpoints as well as informational endpoints will be performed on this PP population. In that case all other analyses will be supportive.

Outcome Definitions

Bleeding Classifications – Bleeding Academic Research Consortium (BARC)[16]

Type 0: No bleeding

Type 1: Bleeding that is not actionable and does not cause the patient to seek unscheduled performance of studies, hospitalization, or treatment by a healthcare professional; may include episodes leading to self-discontinuation of medical therapy by the patient without consulting a healthcare professional.

Type 2: Any overt, actionable sign of hemorrhage (e.g., more bleeding than would be expected for a clinical circumstance, including bleeding found by imaging alone) that does not fit the criteria for type 3, 4, or 5 but does meet at least one of the following criteria: (1) requiring nonsurgical, medical intervention by a healthcare professional, (2) leading to hospitalization or increased level of care, or (3) prompting evaluation.

Type 3a: Overt bleeding plus hemoglobin drop of 3 to <5 g/dL* (provided hemoglobin drop is related to bleed), any transfusion with overt bleeding.

Type 3b: Overt bleeding plus hemoglobin drop ≥5 g/dL* (provided hemoglobin drop is related to bleed), Cardiac tamponade, Bleeding requiring surgical intervention for control (excluding dental/nasal/skin/hemorrhoid), Bleeding requiring intravenous vasoactive agents.

Type 3c: Intracranial hemorrhage (does not include microbleeds or hemorrhagic transformation, does include intraspinal), Subcategories confirmed by autopsy or imaging or lumbar puncture Intraocular bleed compromising vision.

Type 4: CABG-related bleeding, Perioperative intracranial bleeding within 48 h, Reoperation after closure of sternotomy for the purpose of controlling, Bleeding, Transfusion of ≥5 U whole blood or packed red blood cells within a 48-h period, Chest tube output ≥2L within a 24-h period

Type 5: Fatal bleeding

Type 5a: Probable fatal bleeding; no autopsy or imaging confirmation but clinically suspicious

Type 5b: Definite fatal bleeding; overt bleeding or autopsy or imaging confirmation
CABG indicates CABG. Platelet transfusions should be recorded and reported but are not included in these definitions until further information is obtained about the relationship to outcomes. If a CABG-related bleed is not adjudicated as at least a type 3 severity event, it will be classified as not a bleeding event. If a bleeding event occurs with a clear temporal relationship to CABG (i.e., within a 48-h time frame) but does not meet type 4 severity criteria, it will be classified as not a bleeding event.

*Corrected for transfusion (1 U packed red blood cells or 1 U whole blood=1 g/dL hemoglobin).

Bleeding Classifications – GUSTO[17]
According to the GUSTO (Global Use of Strategies to Open Occluded Coronary Arteries) classification of severe, moderate and mild bleeding events:

Severe or life-threatening: Intracranial hemorrhage or bleeding that causes hemodynamic compromise and requires intervention.

Moderate: Bleeding that requires blood transfusion but does not result in hemodynamic compromise.

Mild: Bleeding that does not meet criteria for either moderate or severe bleeding.

Bleeding Classifications – TIMI[18]
Non-CABG related bleeding
Major: Any intracranial bleeding (excluding micro hemorrhages <10 mm evident only on gradient-echo magnetic resonance imaging), clinically overt signs of hemorrhage associated with a drop in hemoglobin of ≥5 g/dL, fatal bleeding (bleeding that directly results in death within 7 d)

Minor: Clinically overt (including imaging), resulting in hemoglobin drop of 3 to <5 g/dL

Requiring medical attention: Any overt sign of hemorrhage that meets one of the following criteria and does not meet criteria for a major or minor bleeding event, as defined above, requiring intervention (medical practitioner-guided medical or surgical treatment to stop or treat bleeding, including temporarily or permanently discontinuing or changing the dose of a medication or study drug), leading to or prolonging hospitalization, or prompting evaluation (leading to an unscheduled visit to a healthcare professional and diagnostic testing, either laboratory or imaging)

Minimal: Any overt bleeding event that does not meet the criteria above

Bleeding in the setting of CABG
Fatal bleeding (bleeding that directly results in death), perioperative intracranial bleeding, reoperation after closure of the sternotomy incision for the purpose of controlling bleeding, transfusion of ≥5 U PRBCs or whole blood within a 48-h period; cell saver transfusion will not be counted in calculations of blood products, chest tube output >2 L within a 24-h period.

Braunwald Classification (see Table S1)

Canadian Cardiovascular Society Angina Classification (**see Table S2)**

Cardiac Death
See Death.

Cardiogenic Shock[22]
Cardiogenic shock is a state of end-organ hypoperfusion due to cardiac failure. The definition of cardiogenic shock includes hemodynamic parameters: persistent hypotension (systolic blood pressure <80 to 90 mm Hg or mean arterial pressure 30 mm Hg lower than baseline) with severe reduction in cardiac index (<1.8 L x min^{-1} x m^{-2} without support or <2.0 to 2.2 L x min^{-1} x m^{-2} with support) and adequate or elevated filling pressure (eg, left ventricular end-diastolic pressure >18 mm Hg or right ventricular end-diastolic pressure >10 to 15 mm Hg)

Clinically Driven Target Lesion Revascularization
Revascularization at the target lesion associated with positive functional ischemia study or ischemic symptoms and an angiographic minimal lumen diameter stenosis ≥50% by quantitative coronary angiography or visual estimate, or revascularization of a target lesion with diameter stenosis ≥70% by quantitative coronary angiography or visual estimate without either angina or a positive functional study.

Clinically Driven Target Vessel Revascularization
Revascularization in the target vessel associated with positive functional ischemia study or ischemic symptoms and an angiographic minimal lumen diameter stenosis ≥50% by quantitative coronary angiography or visual estimate, or revascularization of a target vessel with diameter stenosis ≥70% by quantitative coronary angiography or visual estimate without either angina or a positive functional study.

Death[23]
Cardiac Death: Any death due to proximate cardiac cause (e.g., myocardial infarction, low-output failure, fatal arrhythmia), unwitnessed death and death of unknown cause, and all procedure-related deaths, including those related to concomitant treatment, will be classified as cardiac death.

Vascular Death: Death caused by noncoronary vascular causes, such as cerebrovascular disease, pulmonary embolism, ruptured aortic aneurysm, dissecting aneurysm, or other vascular diseases.

Noncardiovascular Death: Death is defined as any death that is not thought to be the result of a cardiovascular cause. The following categories may be collected: 1. Death resulting from malignancy; 2. Death resulting from pulmonary causes; 3. Death caused by infection (including sepsis); 4. Death resulting from gastrointestinal causes; 5. Death resulting from accident/trauma; 6. Death caused by other noncardiovascular organ failure; 7. Death resulting from other noncardiovascular cause.

Undetermined Death: Undetermined cause of death is defined as a death not attributable to any other category because of the absence of any relevant source documents. Such deaths will be classified as cardiac for end point determination (definition adapted from Garcia-Garcia et al[11]).

Major Adverse Cardiac Events
The composite of cardiac death, myocardial infarction, or target vessel revascularization.

Major Adverse Cardiac And Cerebrovascular Events (MACCE)
A composite of all-cause death, myocardial infarction (Q-wave or non-Q-wave), any clinically driven target lesion revascularization, or stroke.

Myocardial Infarction
Myocardial infarction will be adjudicated according to the third[15] and fourth[12] universal definitions of myocardial infarction, on the basis of the 2010 Academic Research Consortium extended historical definition[13] of myocardial infarction, of Academic Research Consortium-2[11] and using the consideration of the Society for Cardiovascular Angiography and Interventions[14] for a definition of clinically relevant myocardial infarction after coronary revascularization.

Definition of Clinically Relevant Myocardial Infarction After Coronary Revascularization according to Society for Cardiovascular Angiography and Interventions[14]:
1- In patient with normal baseline CK-MB:

- The peak CK-MB measured within 48 hours of the procedure rises to ≥10 x the local laboratory upper limit normal, or to ≥5x upper limit of normal with new pathologic Q waves in ≥2 contiguous leads or new persistent left bundle branch block, OR
- In the absence of CK-MB measurement and a normal baseline cardiac troponin (cTn), a cTn (I or T) level measured within 48 hours of the before percutaneous coronary intervention rises to ≥70 x of the local laboratory upper limit of normal, or
≥35 x upper limit of normal with new pathologic Q waves in ≥2 contiguous leads or new persistent left bundle branch block

2-In patient with elevated baseline CK-MB (or cTn) in whom the biomarker levels are stable or falling: The CK-MB or (cTn) rises by an absolute increment equal to those levels recommended above from the most recent preprocedure level.

3-In patient with elevated CK-MB (or cTn) in whom the biomarker levels have not been shown to be stable or falling:

The CK-MB or (cTn) rises by an absolute increment equal to those levels recommended above, plus new ST-segment elevation or depression, plus signs consistent with a clinically relevant myocardial infarction, such as new onset or worsening heart failure or sustained hypotension.

Definition of Clinically Relevant Myocardial Infarction After Coronary Revascularization according to the Academic Research Consortium-2[11]:
Myocardial infarction

- Absolute rise in cardiac troponin (from baseline) >35 times upper range limit (URL)
- Plus one or more of the criteria below:
    - New significant Q-waves or equivalent*
    - Flow limiting angiographic complications
    - New “substantial” loss of myocardium on imaging

Significant periprocedural myocardial injury:

- Absolute rise in cardiac troponin (from baseline) ≥70 times URL

* Q-wave criteria requires the development of new Q waves ≥40 ms in duration and ≥1 mm deep in voltage in ≥2 contiguous leads.

Third universal definition of myocardial infarction[15]: The term acute myocardial infarction should be used when there is evidence of myocardial necrosis in a clinical setting consistent with acute myocardial ischemia. Under these conditions any one of the following criteria meets the diagnosis for myocardial infarction:

- Detection of rise and/or fall of cardiac biomarkers values [preferably cardiac troponin (cTn) with at least one value above the 99th percentile of the upper reference limit (URL) and with at least one of the following:
  - → Symptoms of ischemia;
  - → New or presumed new significant ST-segment–T wave (ST–T) changes or new left bundle branch block.
  - → Development of pathological Q waves in the ECG;
  - → Imaging evidence of new loss of viable myocardium or new regional wall motion abnormality
  - → Identification of an intracoronary thrombus by angiography or autopsy.
- Cardiac death with symptoms suggestive of myocardial ischemia and presumed new ischemic ECG changes or new left bundle branch block, but death occurred before cardiac biomarkers were obtained, or before cardiac biomarker values would be increased.
- Percutaneous coronary intervention (PCI) related myocardial infarction is arbitrarily defined by elevation of cTn values (>5 x 99th percentile URL) in patients with normal baseline values (≤99th percentile URL) or a rise of cTn values >20% if the baseline values are elevated and are stable or falling. In addition, either
  - → (i) symptoms suggestive of myocardial ischemia or
  - → (ii) new ischemic ECG changes or
  - → (iii) angiographic findings consistent with a procedural complication or
  - → (iv) imaging demonstration of new loss of viable myocardium or new regional wall motion abnormality are required.
- Stent thrombosis associated with myocardial infarction when detected by coronary angiography or autopsy in the setting of myocardial ischemia and with a rise and/or fall of cardiac biomarker values with at least one value above the 99th percentile URL.
- Coronary artery bypass grafting related myocardial infarction is arbitrarily defined by elevation of cardiac biomarker values (>10 x 99th percentile URL) in patients with normal baseline cTn values (≤99th percentile URL). In addition, either
  - → (i) new pathological Q waves or new left bundle branch block, or
  - → (ii) angiographic documented new graft or new native coronary artery occlusion, or
  - → (iii) imaging evidence of new loss of viable myocardium or new regional wall motion abnormality.

Third Universal Definition of Myocardial Infarction (see Table S3)

Fourth universal definition of myocardial infarction[12]

1-The term <u>myocardial injury</u> should be used when there is evidence of elevated cardiac troponin values (cTn) with at least one value above the 99th percentile URL.

The myocardial injury is considered acute if there is a rise and/or fall of cTn values.

2- A<u>cute myocardial infarction: type 1, 2 and 3 myocardial infarction.</u>

Acute myocardial injury with clinical evidence of acute myocardial ischemia and with detection of ris and/or fall of cTn values with at least one value above the 99th percentile URL and at least one of the following:

- → Symptoms of myocardial ischemia;
- → New ischemic ECG changes
- → Development of pathological Q waves in the ECG;
- → Imaging evidence of new loss of viable myocardium or new regional wall motion abnormality in a pattern consistent with an ischemic etiology
- → Identification of a coronary thrombus by angiography or autopsy.

3-Percutaneous coronary intervention (PCI) related myocardial infarction ≤48 h after the index procedure: type 4 and 5 myocardial infarction.

Arbitrarily defined by elevation of cTn values (>5 x 99th percentile URL for type 4a myocardial infarction and >10 x 99th percentile URL for type 5 myocardial infarction) in patients with normal baseline values. Patients with elevated pre-procedural cTn values, in whom the pre-procedural cTn levels are stable (≤20% variation) or falling, must meet the criteria for a >5 or >10-fold increase and manifest a change from the baseline value of >20%. In addition, with at least one of the following:

- → New ischemic ECG changes
- → Development of pathological Q waves in the ECG;
- → Imaging evidence of new loss of viable myocardium or new regional wall motion abnormality in a pattern consistent with an ischemic etiology
- → Angiographic findings consistent with a procedural flow-limiting complication such as coronary dissection, occlusion of a major epicardial artery or graft: side-branch occlusion-thrombus, disruption of collateral flow or distal embolization.

4-Any of the following criteria meets the diagnosis for prior or silent/unrecognized myocardial infarction:

- → Abnormal Q-waves with or without symptoms in the absence of nonischemic causes
- → Imaging evidence of loss of viable myocardium in a pattern consistent with ischemic etiology
- → Patho-anatomical findings of a prior myocardial infarction

Fourth Universal Definition of Myocardial Infarction (see Table S4)

Extended Historical Definition of Myocardial Infarction[13]

According to the guideline myocardial infarction following PCI (Percutaneous Coronary Intervention is defined as follows.

*If biomarkers of myocardial damage (CK and CKMB and Trop <1*URL) and not acute myocardial infarction in progress:*

Periprocedural <48 hours post PCI

A. New pathologic q waves in ≥ 2 contiguous ECG leads **and**:

- any CKMB >1*URL **or**
- in the absence of CKMB: Troponin >1*URL **or**
- in the absence of CKMB and Troponin: CK >1*URL **or**
- in the absence of CKMB and Troponin and CK: CEC decision upon clinical scenario

B. Appropriate cardiac enzyme data (respecting top-down hierarchy, b1 to b3):

b1. CK ≥ 2* URL Confirmed by:

- CKMB >1*URL ***or***
- in the absence of CKMB, Troponin >1*URL ***or***
- in the absence of CKMB and Troponin: CEC decision upon clinical scenario

OR

b2. In the absence of CK: CKMB >3*URL

OR

b3. In the absence of CK and CKMB: Troponin >3*URL

*If baseline biomarkers of myocardial damage: CK and/or CKMB >1*URL or acute myocardial infarction in progress:*

Myocardial infarction, re-infarction (extension) <48 hours post PCI

A. If CK (or CKMB) from index myocardial infarction has not yet reached its maximum level:

- Recurrent thoracic chest pain or ischemia equivalent >20 minutes (or new ECG changes consistent with myocardial infarction)

and

- Appropriate cardiac enzyme data:
  - A rise in CK within 24 hours of the index event >2*URL (confirmed by either CKMB or Troponin >1*URL) and ≥ 50% above the previous level **or**

- In absence of CK: a (post PCI) rise in CKMB within 24 hours of the index event >3*URL and ≥ 50% above the previous level. **or**
- In absence of CK and CKMB: a (post PCI) rise of Troponin within 24 hours of the index event >3*URL and ≥ 50% above the previous level**.**

B. If elevated CK (or CKMB) following the index myocardial infarction has peaked **and** CK level has returned <URL then any new rise in:

- CK >2*URL (confirmed by either CKMB >URL or Troponin >URL) **or**
- in the absence of CK: CKMB >3*URL **or**
- in the absence of CK and CKMB, Troponin >3*URL

C. If CK (or CKMB) following the index myocardial infarction has peaked **and** CK level has NOT returned
to <URL:

- A rise in CK ≥50% above the previous level and >2 URL confirmed by either CKMB >URL or Troponin >URL. **or**
- In absence of CK, when CKMB has NOT returned <URL, a rise in CKMB ≥50% above the previous level and >3 URL. ***or***
- In absence of CK, when CKMB and Troponin has not returned <URL a rise in Troponin ≥ 50% above the previous level and >3*URL

Spontaneous myocardial infarction >48 hours post PCI
A. Recurrent thoracic chest pain or ischemic equivalent **and**

- New pathologic q waves in ≥ 2 contiguous ECG leads **and** any CKMB >1*URL **or**
- in the absence of CKMB: Troponin >1*URL **or**
- in the absence of CKMB and Troponin: CK >1*URL **or**
- in the absence of CKMB and Troponin and CK: CEC decision upon clinical scenario

B. Appropriate cardiac enzyme data (respecting top-down hierarchy):
b1. CK ≥ 2* URL Confirmed by:

- CKMB >1*URL **or**
- in the absence of CKMB: Troponin >1*URL **or**
- in the absence of CKMB and Troponin: CEC decision upon clinical scenario

Or
b2. In the absence of CK: CKMB >3*URL
Or
b3. In the absence of CK and CKMB: Troponin >3*URL
Or
b4. In the absence of CK, CK-MB and Troponin, clinical decision based upon clinical scenario.
URL = upper reference limit, defined as 99th percentile of normal reference range

Definition of Clinically Relevant Myocardial Infarction After Coronary Revascularization[14]:
1- In patient with normal baseline CK-MB:

- The peak CK-MB measured within 48 hours of the procedure rises to ≥10x the local laboratory upper limit of normal, or to ≥5x upper limit of normal with new pathologic Q waves in ≥2 contiguous leads or new persistent left bundle branch block, OR
- In the absence of CK-MB measurement and a normal baseline cTn, a cTn (I or T) level measured within 48 hours of the PCI rises to ≥70x le local laboratory upper limit of normal, OR
- ≥35 upper limit of normal with a new pathologic Q waves in ≥2 contiguous leads or new persistent left bundle branch block,

2-In patient with elevated baseline CK-MB (or cTn) in whom the biomarker levels are stable or falling:
The CK-MB or (cTn) rises by an absolute increment equal to those levels recommended above form the most recent pre-procedure level.

3-In patient with elevated CK-MB (or cTn) in whom the biomarker levels have not been shown to be stable or falling:
The CK-MB or (cTn) rises by an absolute increment equal to those levels recommended above, plus a new ST-segment elevation or depression, plus signs consistent with a clinically relevant myocardial infarction, such as new onset or worsening heart failure or sustained hypotension.

New York Heart Association Classification[24]
Class I: Subjects with no limitation of activities; they suffer no symptoms from ordinary activities.
Class II: Subjects with slight, mild limitation of activity; they are comfortable with rest or with mild exertion.
Class III: Subjects with marked limitation of activity; they are comfortable only at rest.
Class IV: Subjects who should be at complete rest, confined to bed or chair; any physical activity brings on discomfort and symptoms occur at rest

Stent Thrombosis (see Table S5)

Target Lesion Failure
Cardiac death, target vessel myocardial infarction (Q-wave or non-Q-wave), or clinically driven target lesion revascularization.

Target Lesion Revascularization
Repeat percutaneous intervention of the target lesion or bypass surgery of the target vessel performed for restenosis or other complication of target the lesion. The target lesion is defined as the treated segment including the 5 mm margin proximal and distal to the stent.

See also *Clinically Driven Target Lesion Revascularization*.

Target Vessel Failure
Composite end point comprised of cardiac death, target vessel myocardial infarction or clinically driven target vessel revascularization.

Target vessel failure will be reported when any of the following events occur:
- Recurrent myocardial infarction occurs in territory not clearly attributed to a vessel other than target vessel.
- Cardiac death not clearly due to a nontarget vessel end point.
- Target vessel revascularization is determined.

Target Vessel Myocardial Infarction
Myocardial infarction that occurs in a territory that cannot be clearly attributed to a vessel other than the target vessel.

Target Vessel Revascularization
Repeat percutaneous intervention or surgical bypass of any segment of the target vessel.
Target vessel is defined as the entire major coronary vessel proximal and distal to target lesion, including upstream and downstream branches and the target lesion itself.
See also *Clinically Driven Target Vessel Revascularization*.

Table S1. Braunwald Classification[19]

**Severity**		**Clinical circumstances in which unstable angina occurs**		
		**A**	**B**	**C**
		Develops in presence of extra cardiac condition that intensifies myocardial ischemia (secondary unstable angina)	Develops in absence of extra cardiac condition (primary unstable angina)	Develops Within 2 weeks of acute myocardial infarction (post infarction unstable angina)
**I**	New onset of severe angina or accelerated angina; no rest pain	IA	IB	IC
**II**	Angina at rest within past month but not within preceding 48 h (angina at rest, subacute)	IIA	IIB	IIC
**III**	Angina at rest within 48 h (angina at rest, acute)	IIIA	IIIB-$Troponin_{neg}$<br>IIIB-$Troponin_{pos}$	IIIC

Table S2. Canadian Cardiovascular Society Angina Classification[20,21]

Class	Definition
I	Ordinary physical activity does not cause angina such as walking, climbing stairs. Angina (occurs) with strenuous, rapid, or prolonged exertion at work or recreation.
II	Slight limitation of ordinary activity. Angina occurs on walking or climbing stairs rapidly, walking uphill; walking or stair climbing after meals, in cold, in wind, or under emotional stress, or only during the few hours after awakening; Angina occurs on walking more than 2 blocks on the level and climbing more than one flight of ordinary stairs at a normal pace and in normal conditions.
III	Marked limitation of ordinary physical activity. Angina occurs on walking one to two blocks on the level and climbing one flight of stairs in normal conditions and at a normal pace.
IV	Inability to carry on any physical activity without discomfort - angina symptoms may be present at rest.

Table S3. Third Universal Definition of Myocardial Infarction[15]

Classification	Description
Type 1<br>Spontaneous myocardial infarction	Spontaneous myocardial infarction related to atherosclerotic plaque rupture, ulceration, fissuring, erosion, or dissection with resulting intraluminal thrombus in one or more of the coronary arteries leading to decreased myocardial blood flow or distal platelet emboli with ensuing myocyte necrosis. The patient may have underlying severe coronary artery disease but on occasion nonobstructive or no coronary artery disease.
Type 2<br>Myocardial infarction secondary to an ischemic imbalance	In instances of myocardial injury with necrosis where a condition other than coronary artery disease contributes to an imbalance between myocardial oxygen supply and/or demand, e.g. coronary endothelial dysfunction, coronary artery spasm, coronary embolism, tachy-/brady-arrhythmias, anemia, respiratory failure, hypotension and hypertension with or without LVH.
Type 3<br>Myocardial infarction resulting in death when biomarker values are unavailable	Cardiac death with symptoms suggestive of myocardial ischemia and presumed new ischemic ECG changes or new left bundle branch block, but death occurring before blood samples could be obtained, before cardiac biomarker could rise, or in rare cases cardiac biomarkers were not collected.
Type 4a<br>Myocardial infarction related to percutaneous coronary intervention (PCI)	Myocardial infarction associated with PCI is arbitrarily defined by elevation of cTn values >5 x 99th percentile URL in patients with normal baseline values (≤99th percentile URL) or a rise of cTn values >20% if the baseline values are elevated and are stable or falling. In addition, either<br>(i) symptoms suggestive of myocardial ischemia, or<br>(ii) new ischemic ECG changes or new left bundle branch block, or<br>(iii) angiographic loss of patency of a major coronary artery or a side branch or persistent slow or no-flow or embolization, or<br>(iv) imaging demonstration of new loss of viable myocardium or new regional wall motion abnormality are required.
Type 4b<br>Myocardial infarction related to stent thrombosis	Myocardial infarction associated with stent thrombosis is detected by coronary angiography or autopsy in the setting of myocardial ischemia and with a rise and/or fall of cardiac biomarkers values with at least one value above the 99th percentile URL.
Type 5<br>Myocardial infarction related to CABG	Myocardial infarction associated with CABG is arbitrarily defined by elevation of cardiac biomarker values >10 x 99th percentile URL in patients with normal baseline cTn values (£99th percentile URL). In addition, either (i) new pathological Q waves or new left bundle branch block, or (ii) angiographic documented new graft or new native coronary artery occlusion, or (iii) imaging evidence of new loss of viable myocardium or new regional wall motion abnormality.

Table S4. Fourth Universal Definition of Myocardial Infarction[12]

Classification	Description
**Myocardial infarction Type 1**	Detection of a rise and/or fall of cTn values with at least one value above the 99th percentile URL and with at least one of the following:<br>— Symptoms of acute myocardial ischemia<br>— New ischemic ECG changes<br>— Development of pathological Q-waves<br>— Imaging evidence of new loss of viable myocardium or new regional wall motion abnormality in a pattern consistent with an ischemic etiology<br>— Identification of a coronary thrombus by angiography including intracoronary imaging or by autopsy.*<br>*Post-mortem demonstration of an artherothrombus in the artery supplying the infarcted myocardium, or a macroscopically large circumscribed area of necrosis with or without intramyocardial hemorrhage, meets the type 1 myocardial infarction criteria regardless of cTn values.
**Myocardial infarction Type 2**	Detection of a rise and/or fall of cTn values with at least one value above the 99th percentile URL, and evidence of an imbalance between myocardial oxygen supply and demand unrelated to coronary thrombosis, requiring at least one of the following:<br>— Symptoms of acute myocardial ischemia<br>— New ischemic ECG changes<br>— Development of pathological Q-waves<br>— Imaging evidence of new loss of viable myocardium or new regional wall motion abnormality in a pattern consistent with an ischemic etiology
**Type 3 Myocardial infarction resulting in death when biomarker values are unavailable**	Patients who suffer cardiac death with symptoms suggestive of myocardial ischemia accompanied by presumed new ischemic ECG changes or ventricular fibrillation, but die before blood samples for biomarkers can be obtained, or before increase in cardiac biomarkers can be identified, or myocardial infarction is detected by autopsy examination.
**Type 4a Myocardial infarction ≤ 48 h after percutaneous coronary intervention (PCI)**	Coronary intervention-related myocardial infarction is arbitrarily defined by an elevation of cTn values more than five times the 99th percentile URL in patients with normal baseline values. In patients with elevated pre-procedure cTn in whom the cTn level are stable (≤20% variation) or falling, the post-procedure cTn must rise by >20%. However, the absolute post-procedural value must still be at least five times the 99th percentile URL. In addition, one of the following elements is required:<br>— New ischemic ECG changes<br>— Development of new pathological Q-waves*<br>— Imaging evidence of new loss of viable myocardium or new regional wall motion abnormality in a pattern consistent with an ischemic etiology<br>— Angiographic findings consistent with a procedural flow-limiting complication such as coronary dissection, occlusion of a major epicardial artery or a side branch occlusion/thrombus, disruption of collateral flow, or distal embolization.**<br>*Isolated development of new pathological Q-waves meets the type 4a I criteria if cTn values are elevated and rising but more than five times the 99th percentile URL.<br>**Post-mortem demonstration of a procedure-related thrombus in the culprit artery, or a macroscopically large circumscribed area necrosis with or without intra-myocardial hemorrhage meets the type 4a myocardial infarction criteria.

Classification	Description
**Type 4b**<br>**Myocardial infarction related to stent/scaffold thrombosis associated with PCI**	A subcategory of PCI-related myocardial infarction is stent/scaffold thrombosis, type 4b myocardial infarction, as documented by angiography or autopsy using the same criteria utilized for type 1 myocardial infarction. It is important to indicate the time of the occurrence of the stent/scaffold thrombosis in relation to the timing of the PCI procedure. The following temporal categories are suggested:<br>— acute 0-24 h;<br>— subacute >24 h to 30 days;<br>— late >30 days;<br>— very late >1 year after stent/scaffold thrombosis.
**Type 4c Myocardial infarction related to restenosis associated with PCI**	Occasionally myocardial infarction occurs and -at angiography, in-stent restenosis, or restenosis following balloon angioplasty in the infarct territory- is the only angiographic explanation since no other culprit lesion or thrombus can be identified. This PCI-related myocardial infarction type is designated as type 4c myocardial infarction, defined as focal or diffuse restenosis, or a complex lesion associated with a rise and/or fall of cTn values above the 99th percentile URL applying, the same criteria utilized for type 1 myocardial infarction.
**Type 5**<br>**Myocardial infarction related to CABG; ≤48 h after the index procedure**	Myocardial infarction associated with CABG is arbitrarily defined as elevation of cTn values >10 times the 99th percentile URL in patients with normal baseline cTn values. In patients with elevated pre-procedure cTn in whom cTn levels are stable (≤ 20% variation) or falling, the post-procedure cTn must rise by >20%. However, the absolute post-procedural value still must be >10 times the 99th percentile URL. In addition, one of the following elements is required:<br>— Development of new pathological Q-waves*<br>— Angiographic documented new graft occlusion or new native coronary artery occlusion<br>— Imaging evidence of new loss of viable myocardium or new regional wall motion abnormality in a pattern consistent with an ischemic etiology<br>* Isolated development of new pathological Q-waves meets the type 5 myocardial infarction if cTn values are elevated and rising but >10 times the 99th percentile URL.

Table S5. Stent Thrombosis – Academic Research Consortium-2 Definition[11]

Classification	Criteria
Definite stent thrombosis	Angiographic confirmation of stent thrombosis*
	The presence of a thrombus† that originates in the stent or in the segment 5 mm proximal or distal to the stent or in a side branch originating from the stented segment and the presence of at least 1 of the following criteria:
	Acute onset of ischemic symptoms at rest
	New electrocardiographic changes suggestive of acute ischemia
	Typical rise and fall in cardiac biomarkers (refer to definition of spontaneous myocardial infarction)
	Or
	Pathological confirmation of stent thrombosis
	Evidence of recent thrombus within the stent determined at autopsy
	Examination of tissue retrieved following thrombectomy (visual/histology)
Probable stent/scaffold thrombosis	Regardless of the time after the index procedure, any myocardial infarction that is related to documented acute ischemia in the territory of the implanted stent/scaffold without angiographic confirmation of stent/ scaffold thrombosis and in the absence of any other obvious cause.‡
Silent stent occlusion	The incidental angiographic documentation of stent occlusion in the absence of clinical signs or symptoms is not considered stent thrombosis.
Timing of stent thrombosis (duration after stent implantation)	
Acute	0§–24 h
Subacute	>24 h–30 d
Late	>30 d–1 y
Very Late	>1 y

Early stent thrombosis is 0 to 30 days (acute plus subacute stent thrombosis).
*Definite stent thrombosis is considered to have occurred by either angiographic or pathological confirmation.
†Occlusive thrombus: Thrombolysis in Myocardial Infarction grade 0 or 1 flow within or proximal to a stent/scaffold segment. Nonocclusive thrombus: intracoronary thrombus is defined as a (spherical, ovoid, or irregular) noncalcified filling defect or lucency surrounded by contrast material (on 3 sides or within a coronary stenosis) seen in multiple projections, persistence of contrast material within the lumen, or visible embolization of intraluminal material downstream.
‡When the stented segment is in the left circumflex coronary artery or in the presence of preexisting electrocardiographic abnormalities (e.g., left bundle branch block, paced rhythms), definitive evidence of localization may be absent and Clinical Events Committee adjudication is based on review of all available evidence).
§Defined as the moment the patient is undraped and taken off the catheterization table.

Table S6. Procedural Characteristics at the Patient Level[a]

Characteristic	Biodegradable-Polymer Stent (n=969 Patients)	Zotarolimus-Eluting Stent (n=979 Patients)
Access[b], no. (%)		
Radial	815 (84.1)	834 (85.2)
Femoral	137 (14.1)	130 (13.3)
Brachial	10 (1.0)	9 (0.9)
Lesion location[c], no. (%) (at least 1 lesion)		
Left main	45 (3.7)	38 (3.9)
Left anterior descending	529 (55.2)	541 (55.6)
Left circumflex	270 (28.2)	264 (27.1)
Right coronary artery	310 (32.3)	299 (30.7
Bypass graft	16 (1.7)	15 (1.5)
At least one B2/C lesion class[c], no. (%)	583 (60.9)	614 (63.5)
At least one lesion with moderate or severe calcification‡, no. (%)	339 (35.3)	335 (34.5)
At least one lesion with bifurcation[c], no. (%)	290 (30.2)	308 (31.7)
At least one lesion with chronic total occlusion[c], no. (%)	24 (2.5)	22 (2.3)
At least one lesion with in-stent restenosis[c], no. (%)	47 (4.9)	50 (5.1)
Mean reference vessel diameter per subject[c], mm	3.05 ± 0.47	3.05 ± 0.50
Mean diameter stenosis per subject[c], %	82.00 ± 11.79	82.15 ± 13.00
Mean lesion length per subject[c], mm	20.75 ± 11.07	21.25 ± 12.15
Multivessel intervention, no. (%)	210 (21.9)	181 (18.6)
Number of vessels treated per patient[d], no. (%)		
One	738 (77.0)	779 (80.1)
Two	173 (18.0)	150 (15.4)
Three	33 (3.4)	24 (2.5)
Number of stents per patient	1.69 ± 0.998	1.71 ± 0.997
Total stent length per patient	37.15 ± 25.389	36.73 ± 24.432
Any overlapping stenting, no. (%)	174 (18.0)	212 (21.7)

[a] Plus–minus values are means±SD.
[b] Unknown for 7 patients in biodegradable-polymer stent group and 6 patients in the zotarolimus-eluting stent group.
[c] Unknown for 10 patients in biodegradable-polymer stent group and 6 patients in the zotarolimus-eluting stent group (percentages were calculated using a total of 959 patients in the biodegradable-polymer stent group and 973 patients in the zotarolimus-eluting stent group).
[d] Four patients in the biodegradable-polymer stent group and 6 in the zotarolimus-eluting stent group had 4 treated vessels; 1 patient in the zotarolimus-eluting stent group had 5 treated vessels and 11 patient in biodegradable-polymer stent group and 13 in the zotarolimus-eluting stent group had only coronary artery bypass graft treatment.

Table S7. Procedural Characteristics at the Lesion Level[a]

Characteristic	Biodegradable-Polymer Stent (n=1368 Lesions)	Zotarolimus-Eluting Stent (n=1354 Lesions)
Lesion location, no. (%) (at least 1 lesion)		
Left main	45 (3.3)	38 (2.8)
Left anterior descending	617 (45.1)	622 (45.9)
Left circumflex	301 (22.0)	303 (22.4)
Right coronary artery	388 (28.4)	374 (27.6)
Bypass graft, no. (%)	17 (1.2)	17 (1.3)
B2/C lesion class[b], no. (%)	782 (57.1)	779 (57.5)
Moderate or severe calcification, no. (%)	439 (32.1)	426 (31.4)
Bifurcation lesion, no. (%)	329 (24.0)	349 (25.8)
Chronic total occlusion, no. (%)	29 (2.1)	23 (1.7)
In-stent restenosis lesion, no. (%)	55 (4.0)	56 (4.1)
Reference vessel diameter, mm	3.03 ± 0.50	3.03 ± 0.53
Diameter stenosis, %	81.79 ± 13.09	82.15 ± 13.51
Lesion length, mm	20.72 ± 11.75	20.86 ± 12.25

[a] Plus–minus values are means±SD.
[b] Lesion class was unknown for 4 lesions in biodegradable-polymer stent group and 9 lesions in zotarolimus-eluting stent group.

Table S8. Characteristics of Target Lesions[a]

Characteristic	Biodegradable-Polymer Stent (n=969 Patients)	Zotarolimus-Eluting Stent (n=979 Patients)
Number of target lesions	1368	1354
Lesion location, no. (%)		
Left main	45 (3.3)	38 (2.8)
Left arterial descending artery	617 (45.1)	622 (45.9)
Left circumflex artery	301 (22.0)	303 (22.4)
Right coronary artery	388 (28.4)	374 (27.6)
Bypass graft, no. (%)	17 (1.2)	17 (1.3)
Bifurcation, no. (%)	329 (24.0)	349 (25.8)
TIMI flow before PCI per lesion, no. (%)		
0 or 1	71 (5.2)	65 (4.8)
2	86 (6.3)	98 (7.2)
3	1211 (88.5)	1190 (87.9)
Total number of lesions treated, no. (%)	1366	1349
Lesions treated with balloon	8 (0.6)	7 (0.5)
Lesions treated with stent implantation	1358 (98.5)	1342 (98.7)
Number of stented lesions, no. (%)	n=1358	n=1342
Lesions treated with study stent only	1347 (99.2)	1338 (99.8)
Lesions treated with non-study stent only	8 (0.6)	2 (0.1)
Lesions implanted with study stent + non-study stent	3 (0.2)	2 (0.1)
Total number of implanted stents, no. (%)	1626	1662
Study stent	1612	1657
Other drug-eluting stent	14	5
Number of study stents implanted per lesion	1350<br>1.19 ± 0.47	1341<br>1.24 ±0.51
Multiple study stents implanted (lesion level), no. (%)	N=222	N=272
Overlapping stenting	181 (13.3)	222 (16.5)
Implanted stent length, mm *(device level)*	21.62 ± 8.20	21.33 ± 8.29
Implanted stent diameter, mm *(device level)*	2.99 ± 0.47	3.00 ± 0.49
Direct stenting per lesion, no. (%)	260 (19.1)	284 (21.2)
Post-dilatation per lesion, no. (%)	711 (47.3)	698 (52.0)
Final residual stenosis, (%)	1.35 ± 5.3	1.46 ± 6.6
Device success, defined as attainment of <30% residual stenosis of the target lesion using the assigned study stents only, no. (%)	N=1636[b]<br>1589 (97.1)	N=1670[b]<br>1629 (97.9)

[a] Number (%). Plus–minus values are means±SD.
[b] Number of study stents opened (implanted and non-implanted).
PCI, percutaneous coronary intervention; TIMI, Thrombolysis In Myocardial Infarction.

Table S9. Medications

**Medication**	**Biodegradable-Polymer Stent**	**Zotarolimus-eluting Stent**
**At day 1, no. (%)**	**(n=956)[a]**	**(n=968)[a]**
Dual antiplatelet therapy	921 (97.0)	950 (98.1)
Single antiplatelet therapy	23 (2.9)	10 (1.0)
No antiplatelet therapy	12 (1.2)	8 (0.8)
Aspirin	928 (97.0)	953 (98.4)
$P2Y_{12}$ inhibitor	937 (98.1)	957 (98.8)
Clopidogrel	841 (89.7)	869 (90.8)
Prasugrel	6 (0.6)	7 (0.7)
Ticagrelor	90 (9.6)	81 (8.4)
Oral anticoagulant	317 (33.2)	360 (37.1)
Statin	733 (76.6)	777 (80.2)
**On day 30, no. (%)**	**(n=934)[a]**	**(n=961)[a]**
Dual antiplatelet therapy	769 (82.3)	807 (83.9)
Single antiplatelet therapy	162 (17.3)	150 (15.6)
No antiplatelet therapy	3 (0.3)	4 (0.4)
Aspirin	832 (89.0)	870 (90.5)
$P2Y_{12}$ inhibitor	868 (92.9)	894 (93.0)
Clopidogrel	784 (90.3)	812 (90.8)
Prasugrel	6 (0.6)	6 (0.6)
Ticagrelor	78 (8.9)	76 (7.5)
Oral anticoagulant	333 (35.6)	381 (39.6)
Statin	754 (80.7)	802 (83.4)
**On day 180, no. (%)**	**(n=912)[a]**	**(n=928)[a]**
Dual antiplatelet therapy	42 (4.6)	36 (3.8)
Single antiplatelet therapy	833 (91.3)	848 (91.3)
No antiplatelet therapy	37 (4.1)	44 (4.7)
Aspirin	414 (45.4)	435 (46.8)
$P2Y_{12}$ inhibitor	503 (55.1)	485 (52.2)
Clopidogrel	448 (89.0)	435 (89.6)
Prasugrel	5 (1.0)	7 (1.4)
Ticagrelor	50 (9.1)	43 (8.8)
Oral anticoagulant	339 (37.1)	375 (40.4)
Statin	745 (81.6)	780 (84.1)
**On day 365, no. (%)**	**(n=594)[a]**	**(n=604)[a]**
Dual antiplatelet therapy	24 (4.0)	22 (3.6)
Single antiplatelet therapy	441 (74.2)	457 (75.6)
No antiplatelet therapy	129 (21.7)	125 (20.7)
Aspirin	271 (45.6)	275 (45.5)
$P2Y_{12}$ inhibitor	218 (36.7)	226 (37.4)
Clopidogrel	193 (88.5)	203 (89.8)

Medication	Biodegradable-Polymer Stent	Zotarolimus-eluting Stent
Prasugrel	5 (2.2)	2 (0.8)
Ticagrelor	20 (9.1)	21 (9.2)
Oral anticoagulant	234 (39.4)	252 (41.7)
Statin	482 (81.1)	501 (82.9)

[a] Patients in whom medications were assessed.

Table S10. Additional Information on Type of Antiplatelet Therapy After Randomization

Medication	Biodegradable-Polymer Stent	Zotarolimus-eluting Stent
**At day 1, no. (%)**	**(n=956)[a]**	**(n=968)[a]**
Dual antiplatelet therapy	921 (96.3)	950 (98.1)
Clopidogrel	826 (86.4)	863 (89.1)
Prasugrel	6 (0.6)	7 (0.7)
Ticagrelor	89 (9.3)	80 (8.3)
Single antiplatelet therapy	23 (2.4)	10 (1.0)
Aspirin	7 (0.7)	3 (0.3)
Clopidogrel	15 (1.6)	6 (0.6)
Prasugrel	0 (0.0)	0 (0.0)
Ticagrelor	1 (0.1)	1 (0.1)
Unknown or no medication	12 (1.3)	8 (0.8)
**At day 30, no. (%)**	**(n=934)[a]**	**(n=961)[a]**
Dual antiplatelet therapy	769 (82.3)	807 (84.0)
Clopidogrel	699 (74.8)	733 (76.2)
Prasugrel	4 (0.4)	6 (0.6)
Ticagrelor	66 (7.1)	68 (7.1)
Single antiplatelet therapy	162 (17.3)	150 (15.6)
Aspirin	63 (6.7)	63 (6.6)
Clopidogrel	85 (9.1)	79 (8.2)
Prasugrel	2 (0.2)	0 (0.0)
Ticagrelor	12 (1.2)	8 (0.8)
Unknown or no medication	3 (0.3)	4 (0.4)
**At day 180, no. (%)**	**(n=912)[a]**	**(n=928)[a]**
Dual antiplatelet therapy	42 (4.6)	36 (3.8)
Clopidogrel	36 (3.9)	32 (3.4)
Prasugrel	1 (0.1)	1 (0.1)
Ticagrelor	5 (0.5)	3 (0.3)
Single antiplatelet therapy	833 (91.3)	848 (91.3)
Aspirin	372 (40.1)	399 (43.0)
Clopidogrel	412 (45.2)	403 (43.4)
Prasugrel	4 (0.4)	6 (0.6)
Ticagrelor	45 (4.9)	40 (4.3)
Unknown or no medication	37 (4.05)	44 (4.7)
**At day 365, no. (%)**	**(n=594)[a]**	**(n=604)[a]**
Dual antiplatelet therapy	24 (4.0)	22 (3.6)
Clopidogrel	21 (3.5)	20 (3.3)
Prasugrel	1 (0.2)	0 (0.0)
Ticagrelor	2 (0.3)	2 (0.3)
Single antiplatelet therapy	441 (74.2)	457 (75.6)
Aspirin	247 (41.6)	253 (41.9)

Medication	Biodegradable-Polymer Stent	Zotarolimus-eluting Stent
Clopidogrel	172 (29.0)	183 (30.3)
Prasugrel	4 (0.7)	2 (0.3)
Ticagrelor	18 (3.0)	19 (3.1)
Unknown or no medication	129 (21.7)	125 (20.7)

[a] Patients in whom medications were assessed.

Table S11. Primary and Secondary Outcomes in the Intention-To-Treat Population of Patients with Full Source Data Verification Versus Patients without Full source Data Verification

Outcome	Hazard Ratio (95% CI)
Any revascularization	1.206 (0.770, 1.889)
Bleeding, BARC	
Type 2	1.047 (0.725, 1.512)
Type 2-5	1.032 (0.759, 1.404)
Type 3 or 5	1.076 (0.639, 1.813)
Type 4	Not applicable
Type 5	1.710 (0.286,10.234)
Bleeding, general question (type 1 to 5 - any bleeding)	1.003 (0.771, 1.306)
Cardiac death (including unknown)	0.643 (0.281, 1.473)
Clinically driven target lesion revascularization	0.549 (0.228, 1.327)
Clinically driven target vessel revascularization	0.904 (0.481, 1.701)
Definite and probable stent thrombosis	2.214 (0.744, 6.586)
Definite stent thrombosis	2.587 (0.749, 8.935)
Death - All causes	1.143 (0.707, 1.849)
Bleeding, GUSTO	
Moderate	1.119 (0.584, 2.145)
Moderate or severe	0.975 (0.558, 1.703)
Severe	0.605 (0.204, 1.799)
Hemorrhagic stroke	Not applicable
Ischemic stroke	1.613 (0.528, 4.931)
MACCE (myocardial infarction according to Academic Research Consortium-2)	1.047 (0.698, 1.569)
MACCE (myocardial infarction according to third universal definition)	1.081 (0.780, 1.499)
Myocardial infarction	
Academic Research Consortium-2 definition	0.546 (0.186, 1.604)
General question	1.110 (0.707, 1.742)
Third universal definition	1.110 (0.707, 1.742)
Net adverse cardiovascular events	1.026 (0.732, 1.439)
Noncardiovascular death	1.715 (0.911, 3.227)
Primary outcome (myocardial infarction according to Academic Research Consortium-2)	0.774 (0.435, 1.378)
Additional primary outcome (myocardial infarction according to third universal definition)	0.912 (0.606, 1.374)
Probable stent thrombosis	1.280 (0.116,14.121)
Stroke	1.173 (0.407, 3.375)
Early definite + probable stent thrombosis	2.595 (0.524,12.859)
Late definite + probable stent thrombosis	1.922 (0.430, 8.586)
Bleeding, TIMI	
Minor	0.954 (0.462, 1.971)
Minor or major	0.902 (0.491, 1.657)
Major	0.643 (0.215, 1.923)
Target lesion failure (target vessel myocardial infarction according to Academic Research Consortium-2)	0.557 (0.318, 0.973)
Target lesion failure (target vessel myocardial infarction according to third universal definition)	0.710 (0.464, 1.087)
Target vessel failure (target vessel myocardial infarction according to Academic Research Consortium-2)	0.700 (0.432, 1.134)
Target vessel failure (target vessel myocardial infarction according to third universal definition)	0.793 (0.534, 1.179)

Outcome	Hazard Ratio (95% CI)
Target vessel myocardial infarction (Academic Research Consortium-2)	0.410 (0.121, 1.384)
Target vessel myocardial infarction (third universal definition)	0.971 (0.588; 1.603)

BARC, Bleeding Academic Research Consortium; USTO, Global Use of Strategies to Open Occluded Coronary Arteries; MACCE, major adverse cardiac and cerebrovascular events TIMI = Thrombolysis In Myocardial Infarction.

Figure S1. Flow Diagram

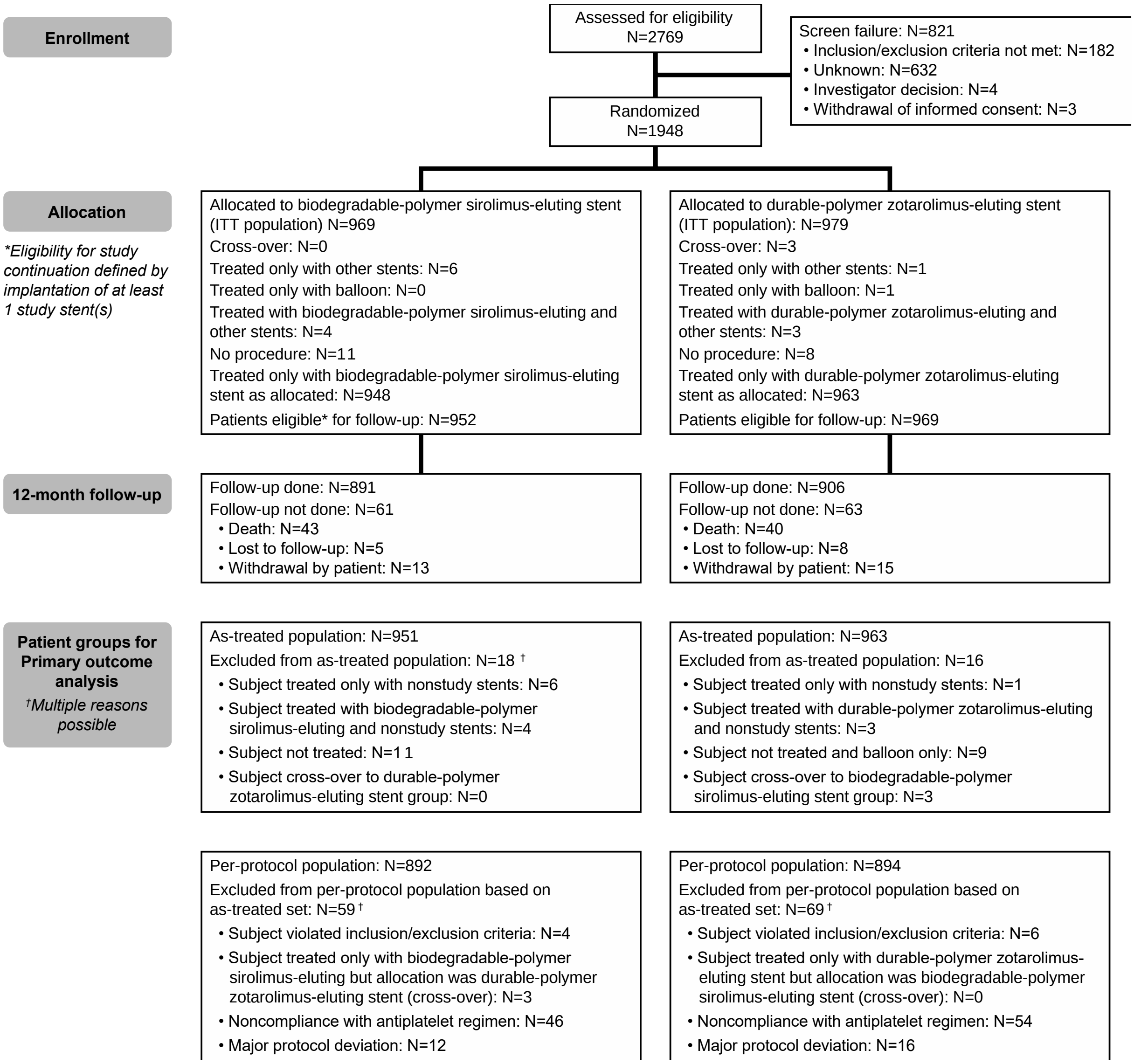

Figure S2. Adherence to Antiplatelet Therapy

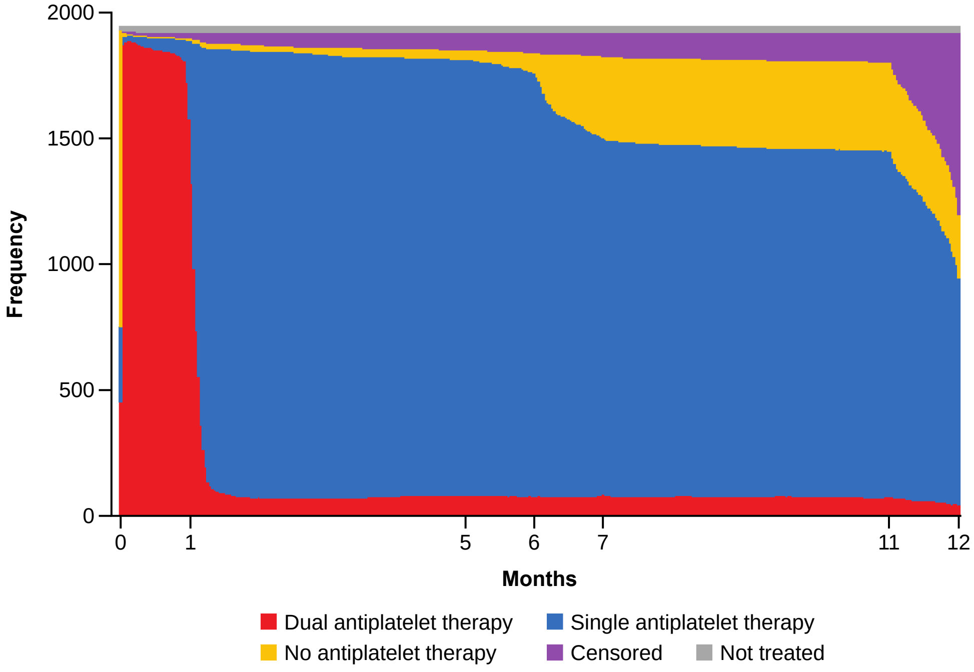

Figure S3. Incidence of the Composite Primary Outcome (Academic Research Consortium-2 Definition) in Prespecified Subgroups

Subgroup	Biodegradable-Polymer Stent Events/N (%)		Zotarolimus-eluting Stent Events/N (%)		Riskdiff (95% CI)
**Age**					
≤ 65 years	3/103	(2.9)	1/118	(0.8)	2.07 (-1.58, 5.71)
> 65 years	30/807	(3.7)	31/811	(3.8)	-0.10 (-1.96, 1.75)
**Gender**					
Male	24/612	(3.9)	22/646	(3.4)	0.52 (-1.56, 2.59)
Female	9/298	(3.0)	10/283	(3.5)	-0.51 (-3.41, 2.39)
**Patients with small vessel(s)**					
≤ 2.75 mm	14/394	(3.6)	11/396	(2.8)	0.78 (-1.67, 3.22)
> 2.75 mm	19/514	(3.7)	21/532	(3.9)	-0.25 (-2.57, 2.07)
**Diabetes**					
Diabetes	7/281	(2.5)	13/294	(4.4)	-1.93 (-4.90, 1.04)
No diabetes	26/629	(4.1)	19/635	(3.0)	1.14 (-0.90, 3.18)
**Renal function**					
No	14/654	(2.1)	20/670	(3.0)	-0.84 (-2.54, 0.86)
Yes	19/256	(7.4)	12/258	(4.7)	2.77 (-1.34, 6.88)
**Patients with long lesion(s)**					
≤ 26 mm	21/649	(3.2)	21/662	(3.2)	0.06 (-1.84, 1.97)
> 26 mm	12/259	(4.6)	11/266	(4.1)	0.50 (-3.01, 4.00)
**Patients with multiple stents in lesion(s)**					
Single stent	16/703	(2.3)	19/677	(2.8)	-0.53 (-2.19, 1.13)
Multiple stents	17/207	(8.2)	13/252	(5.2)	3.05 (-1.58, 7.68)
**Bleeding risk score**					
Precise DAPT score < 25	20/678	(2.9)	19/710	(2.7)	0.27 (-1.47, 2.01)
Precise DAPT score ≥ 25	13/232	(5.6)	13/218	(6.0)	-0.36 (-4.68, 3.96)
**Planned single antiplatelet therapy**					
ASA	15/440	(3.4)	15/451	(3.3)	0.08 (-2.29, 2.45)
P2Y12	18/470	(3.8)	17/478	(3.6)	0.27 (-2.13, 2.67)
**ACS at Baseline**					
No	9/312	(2.9)	12/302	(4.0)	-1.09 (-3.97, 1.79)
Yes	24/596	(4.0)	20/627	(3.2)	0.84 (-1.26, 2.93)
**Acute MI at Baseline**					
No	17/535	(3.2)	18/556	(3.2)	-0.06 (-2.15, 2.03)
Yes	10/187	(5.3)	8/189	(4.2)	1.11 (-3.20, 5.43)

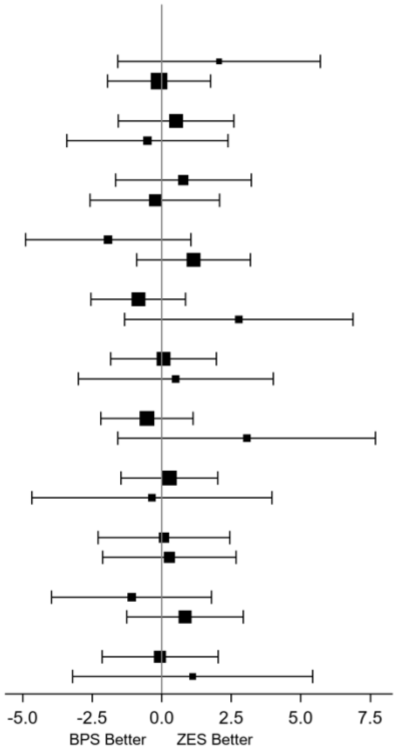

Figure S4. Cumulative Incidence of the Composite Primary Outcome (Third Universal Definition)

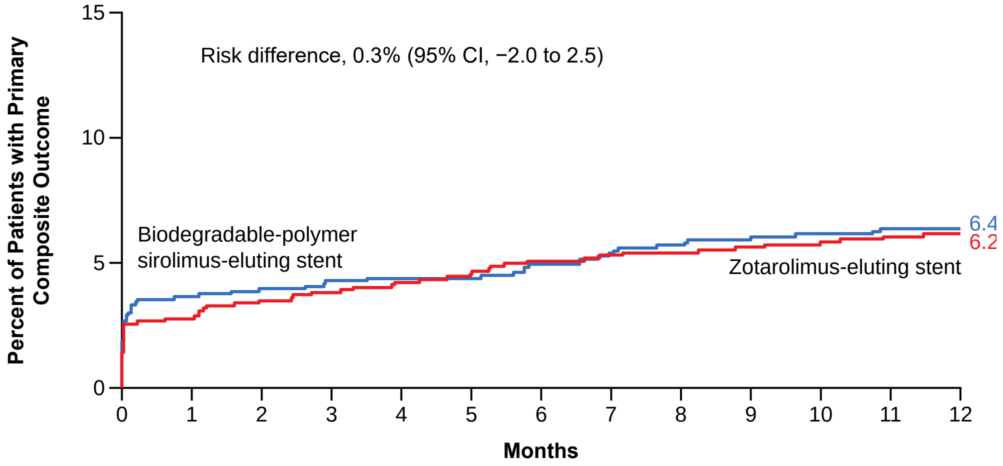

No. at Risk

	0	1	6	12
Biodegradable-polymer sirolimus-eluting stent	934	905	873	510
Zotarolimus-eluting stent	954	936	892	527

Figure S5. Cumulative Incidence of the Composite Primary Outcome (Academic Research Consortium-2 Definition) in a Landmark Analysis

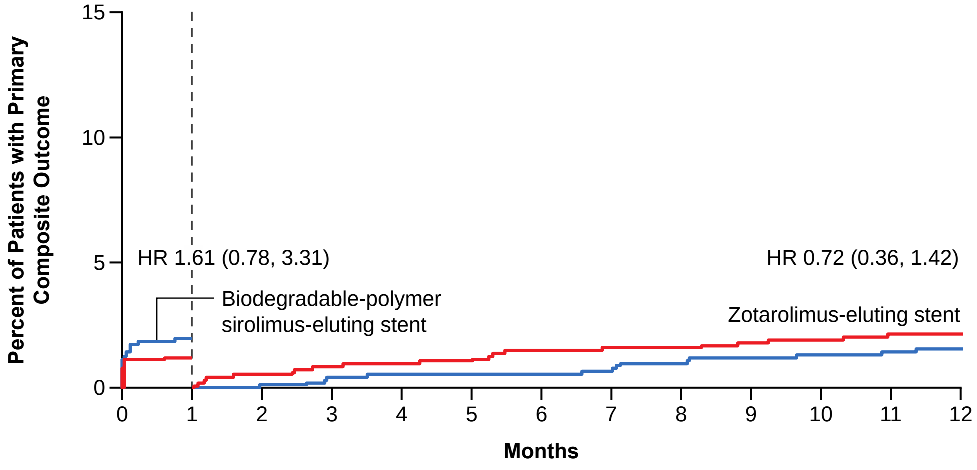

No. at Risk

Biodegradable-polymer sirolimus-eluting stent	941	921 (920 at day 31)	896	526
Zotarolimus-eluting stent	960	950 (949 at day 31)	913	537

Figure S6. Cumulative Incidence of (A) Death from Cardiac Causes; (B) Myocardial Infarction (Academic Research Consortium-2 Definition); and (C) Definite or Probable Stent Thrombosis in a Landmark Analysis

A

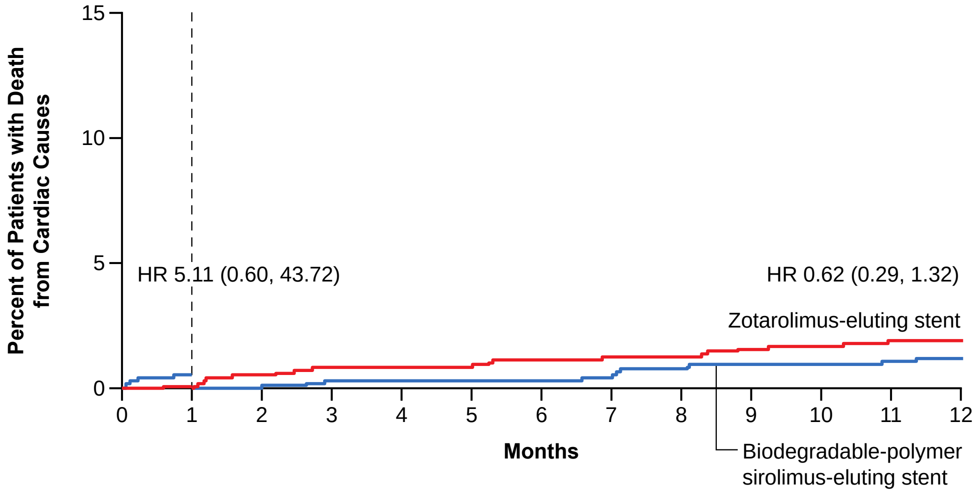

No. at Risk

Biodegradable-polymer sirolimus-eluting stent	952	934 (933 at day 31)	911	536
Zotarolimus-eluting stent	968	961 (960 at day 31)	927	547

B

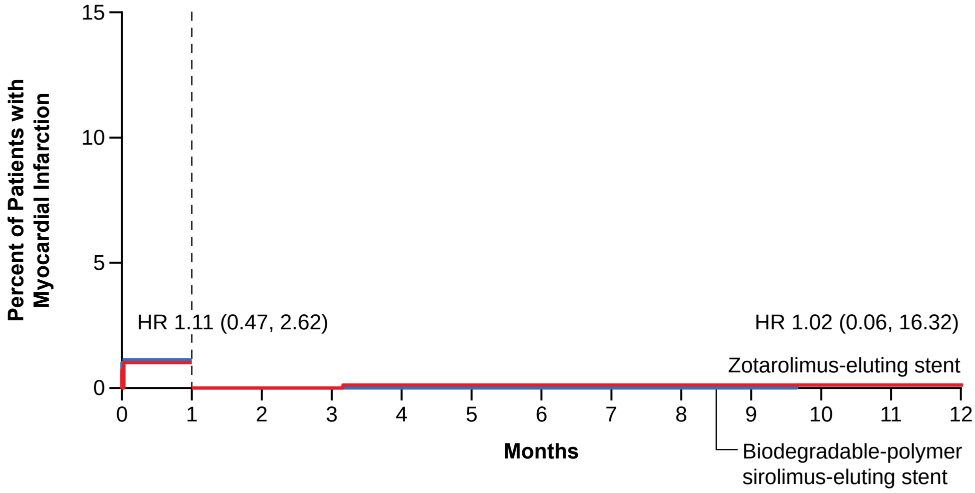

No. at Risk

Biodegradable-polymer sirolimus-eluting stent	942	924 (923 at day 31)	901	528
Zotarolimus-eluting stent	961	951 (950 at day 31)	917	540

C

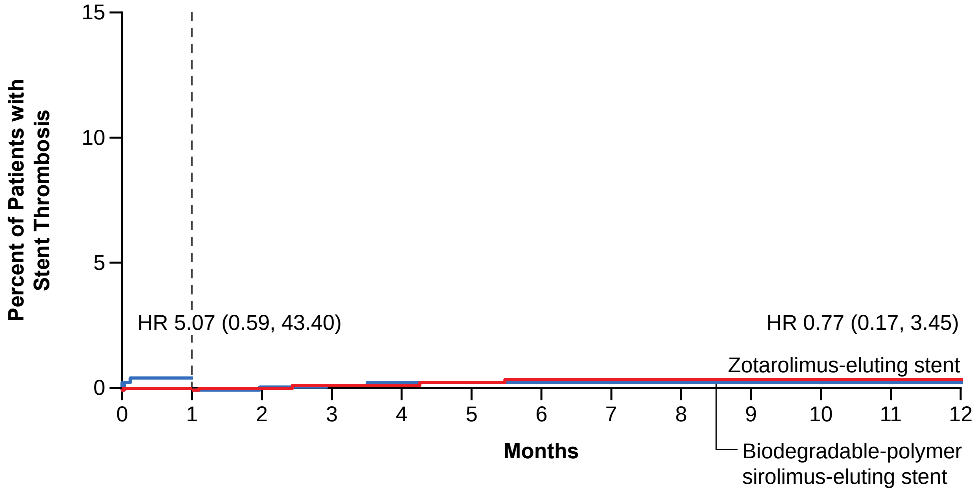

No. at Risk

Biodegradable-polymer sirolimus-eluting stent	949	929 (928 at day 31)	904	533
Zotarolimus-eluting stent	967	960 (959 at day 31)	923	544

Figure S7. Cumulative Incidence of Target Lesion Failure in a Landmark Analysis

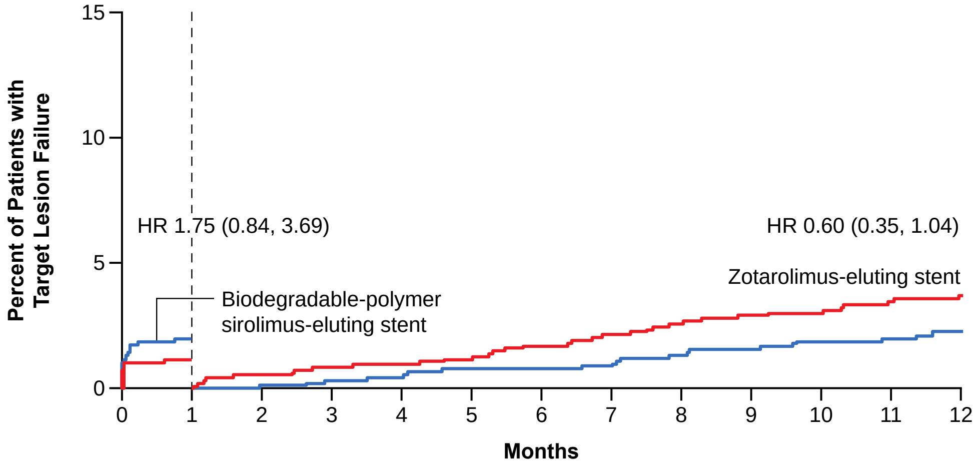

No. at Risk

Biodegradable-polymer sirolimus-eluting stent	942	921 (920 at day 31)	894	522
Zotarolimus-eluting stent	961	951 (950 at day 31)	912	532

Figure S8. Cumulative Incidence of Target Vessel Failure in a Landmark Analysis

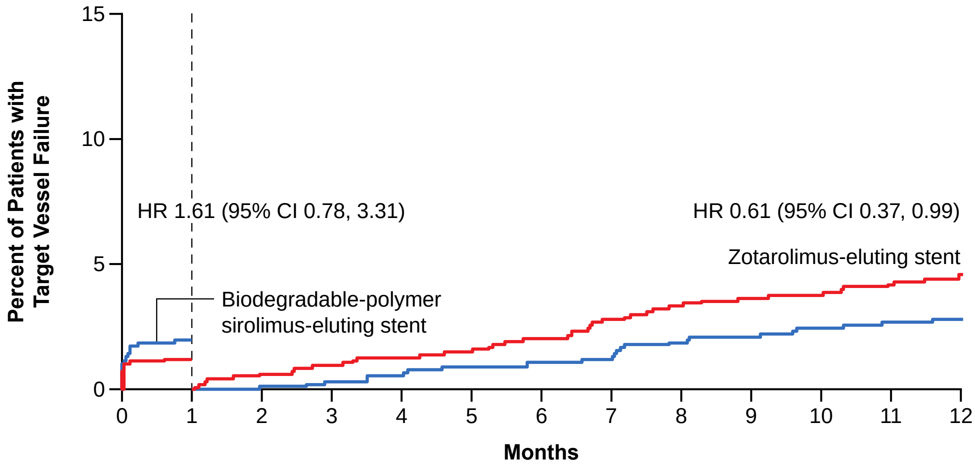

**No. at Risk**				
Biodegradable-polymer sirolimus-eluting stent	942	921 (920 at day 31)	891	518
Zotarolimus-eluting stent	961	950 (949 at day 31)	909	527

BIOFLOW-DAPT Trial: Committees and Investigators

Coordinating Principal Investigator

Marco Valgimigli, MD, PhD	Cardiocentro Ticino Institute, Ente Ospedaliero Cantonale, Lugano, Switzerland

Executive Committee

A. Wlodarczak, MD	Miedziowe Centrum Zdrowia SA, Lubin, Poland
R. Tölg, MD	Bad Segeberger Kliniken GmbH, Germany
B. Merkely, MD	Semmelweis Medical University, Budapest, Hungary
M. Moccetti, MD	Cardiocentro Ticino Institute, Lugano, Switzerland
H. Kelbæk, MD	Sjaellands Universitets Hospital, Roskilde, Denmark
G. Cayla, MD	CHU de Nîmes, Frances

Steering Committee

J. Legutko, MD	John Paul II Hospital, Poland
S. Galli, MD	Centro Cardiologico Monzino, Italy
M. Godin, MD	Clinique St. Hilaire, France
G. Toth-Gayor, MD	Medizinische Universität Graz, Austria
T. Lhermusier, MD	Centre Hospitalier de Rangueil, France
B. Honton, MD	Clinique Pasteur, Toulouse, France
F. Sanguineti	Hôpital Privé / Institut Jacques Cartier, France
P. L. Dietrich, MD	Stadtspital Triemli, Zürich, Switzerland
F. Stammen, MD	AZ Delta Roeselare-Menen, Belgium
B. Ferdinande, MD	Ziekenhuis Oost-Limburg, Belgium
J. Silvain, MD	Hopital Pitie-Salpetriere, France
D. Capodanno, MD	Policlinico G. Rodolico-San Marco, Catania, Italy

Sponsor
BIOTRONIK AG, Vascular Intervention, Ackerstrasse 6, 8180 Bülach, Switzerland

Data Monitoring Committee
Prof. Ralf Birkemeyer (CEC chairman)
78727 Oberndorf, Germany

Steffen Schneider, PhD (DSMB Member)
Institut für Herzinfarktforschung, 67063 Ludwigshafen, Germany

Priv.-Doz. Dr. Lutz Büllesfeld
St Marien Hospital Bonn, 53115 BONN, Germany

Priv.-Doz. Dr. Bernhard Witzenbichler
Helios Amper-Klinikum Dachau, 85221 Dachau, Germany

Independent Statistical Analysis
Dr. Matthias Hochadel
Institut für Herzinfarktforschung, 67063 Ludwigshafen, Germany

Countries, Investigators, and Numbers of Patients Enrolled

Country	Principal Investigator	Site	Patients Enrolled (N=1948)	Patients Screened not Randomized (N=821)
**AUSTRALIA**			**32**	**24**
	N. Collins, MD	John-Hunter Hospital, New Lambton Heights	21	10
	I. Shiekh, MD	Royal Perth Hospital	10	10
	W. Van Gaal, MD	Northern Hospital, Epping	1	4
**AUSTRIA**			**84**	**45**
	G. Toth-Gayor, MD	Medizinische Universitaet Graz	65	12
	M-C. Brandt, MD	Uniklinikum Salzburg	19	33
**BELGIUM**			**111**	**4**
	F. Stammen, MD	AZ Delta Roeselare	46	2
	B. Ferdinande, MD	Ziekenhuis Oost Limburg Genk (ZOL Genk)	44	1
	J. Kefer, MD	Cliniques Universitaires Saint-Luc (UCL)	12	0
	P. Coussement, MD	AZ Sint-Jan Brugge	9	1
**DENMARK**			**152**	**45**
	H. Kelbæk, MD	Sjaellands Universitets Hospital, Roskilde	126	20
	N.-T. Olsen, MD	Gentofte University Hospital	26	25
**FRANCE**			**418**	**23**
	G. Cayla, MD	CHU de Nîmes	100	0
	M. Godin, MD	Clinique Saint Hilaire	69	4
	T. Lhermusier, MD	Clinique Pasteur, Toulouse	58	3
	B. Honton, MD	Hôpital Rangueil - Center Hospitalier de Toulouse	58	1
	F. Sanguineti	Hôpital Privé / Institute Jacques Cartier	51	1
	J. Silvain, MD	Hopital Pitie-Salpetriere	43	2
	E. Puymirat, MD	Hôpital European Georges Pompidou	25	1
	G. Lemesle, MD	CHRU de Lille	8	10
	D. Romain, MD	CHU de Brest	6	1
**GERMANY**			**218**	**388**
	R. Tölg, MD	Segeberger Kliniken	166	182
	M. Haude, MD	Lukaskrankenhaus Neuss	25	102
	T. Schmitz, MD	Elisabeth-Krankenhaus Essen	12	55
	M. Sherif, MD	Universitätsklinikum der Charité, Campus Virchow-Klinikum	11	47
	J. Wöhrle, MD	Klinikum Friedrichshafen GmbH	4	2
**HONG KONG**			**12**	**16**
	A. Yung, MD	Queen Mary Hospital	9	11
	B. Yan, MD	Prince of Wales Hospital	3	5
**HUNGARY**			**177**	**2**
	B. Merkely, MD	University of Semmelweis	152	2
	A. Vorobcsuk, MD	Moritz Kaposi General Hospital	17	0
	I. Horvath, MD	The University of Pécs	8	0
**ITALY**			**141**	**72**

Country	Principal Investigator	Site	Patients Enrolled (N=1948)	Patients Screened not Randomized (N=821)
	S. Galli, MD	Centro Cardiologico Monzino	82	1
	D. Capodanno, MD	Azienda Ospedaliero - Ferrarotto Alessi	31	46
	M. Ferlini, MD	Fondazione Irccs Policlinico San Matteo	25	24
	E. Nicolini, MD	Azienda Ospedaliero Universitaria Ospedali Riuniti Umberto I - G.M. Lancisi	3	1
**LATVIA**			**22**	**2**
	A. Erglis, MD	Pauls Stradins Clinical University Hospital	16	1
	A. Kalnins, MD	Riga East Clinical University Hospital	6	1
**MALAYSIA**			**3**	**9**
	S. Azmi, MD	Institute Jantung Negara	3	9
**NETHERLAND**			**1**	**0**
	S. Somi, MD	HagaZiekenhuis	1	0
**NEW ZEALAND**			**8**	**21**
	J. Somaratne, MD	Auckland City Hospital	8	21
**POLAND**			**299**	**1**
	A. Wlodarczak, MD	Miedziowe Centrum Zdrowia SA (Copper Center)	201	0
	J. Legutko, MD	Krakowski Szpital Specjalistyczny / John Paul II Hospital	98	1
**SINGAPORE**			**1**	**6**
	D. Khoo Zhi Lin, MD	Tan Tock Seng Hospital - Cardiology	1	6
**SPAIN**			**14**	**0**
	J. M. De La Torre Hernandez, MD	Hospital Universitario Marques de Valdecilla	12	0
	A. Torres Bosco, MD	Hospital Clínico de Valencia	1	0
	J. Sanchis Fores, MD	Hospital Universitario de Araba	1	0
**SWITZERLAND**			**232**	**160**
	M. Moccetti, MD	Fondazione Cardiocentro Ticino	132	115
	P. L. Dietrich, MD	Stadtspital Triemli	50	35
	S. Fournier, MD	Centre Hospitalier Universitaire Vaudoise	20	8
	J. Iglesias, MD	Hôpitaux Universitaires Genève (HUG)	20	0
	V. Rubimbura, MD	Hôpital de Morges	10	2
**THAILAND**			**23**	**3**
	W. Kehasukcharoen, MD	Central Chest Institute of Thailand	13	0
	S. Nakarin, MD	Phramongkutklao Hospital	10	3

BIOFLOW-DAPT Trial Investigators

Marco Valgimigli, MD, PhD, Cardiocentro Ticino Institute, Ente Ospedaliero Cantonale (EOC), Università della Svizzera Italiana, CH-6900, Lugano, Switzerland

Adrian Wlodarczak, MD, Poland Miedziowe Centrum Zdrowia Lubin, Lubin, Poland

Ralph Tölg, MD, Herzzentrum der Segeberger Kliniken GmbH, Bad Segeberg, Germany

Béla Merkely, MD, Semmelweis University, Heart and Vascular Center, Budapest, Hungary

Marco Moccetti, Cardiocentro Ticino, Via Tesserete 48, 6900 Lugano, Switzerland

Henning Kelbæk, MD, Department of Cardiology, Zealand University Hospital, Sygehusvej 10, 4000 Roskilde, Denmark

Jacek Legutko, MD, Department of Interventional Cardiology, Institute of Cardiology, Jagiellonian University Medical College, John Paul II Hospital, Krakow, Poland

Stefano Galli, MD, Department of Interventional Cardiology, Centro Cardiologico Monzino, Istituto di Ricovero e Cura a Carattere Scientifico, Milan, Italy

Matthieu Godin, MD, Department of Cardiology, Clinique Saint Hilaire, 2 place Saint Hilaire 76000 Rouen, France

Gabor G. Toth-Gayor, MD, University Heart Center Graz, Department of Cardiology, Medical University Graz, Graz, Austria

Thibault Lhermusier, MD, Hôpital de Rangueil, Fédération de Cardiologie, Pôle Cardio-vasculaire et Métabolique, Toulouse, France

Benjamin Honton, MD, Department of Interventional Cardiology, Clinique Pasteur, Toulouse, France

Francesca Sanguineti, Hôpital Privé / Institut Jacques Cartier, France

Peter Laurenz Dietrich, MD, Stadtspital Triemli, Zürich, Switzerland

Francis Stammen, MD, Department of Cardiology, AZ Delta, Roeselare, Belgium

Bert Ferdinande, MD, Department of Cardiology, Hospital Oost-Limburg Genk, Belgium

Johanne Silvain, MD, Sorbonne Université, ACTION Group, INSERM UMRS 1166, Hôpital Pitié-Salpêtrière (AP-HP), Institut de Cardiologie, Paris, France

Davide Capodanno, MD, Division of Cardiology, Azienda Ospedaliero Universitaria Policlinico "G. Rodolico-San Marco", University of Catania, Via Santa Sofia, 78, Catania 95123, Italy

Ralf Birkemeyer (CEC chairman), 78727 Oberndorf, Germany

Steffen Schneider, PhD (DSMB Member), Institut für Herzinfarktforschung, 67063 Ludwigshafen, Germany

Lutz Büllesfeld, St Marien Hospital Bonn, 53115 Bonn, Germany

Bernhard Witzenbichler, Helios Amper-Klinikum Dachau, 85221 Dachau, Germany

Matthias Hochadel, Institut für Herzinfarktforschung, 67063 Ludwigshafen, Germany

Nicolas Collins, MD, John-Hunter Hospital, New Lambton Heights, Australia

Imran Shiekh, MD, Royal Perth Hospital, Australia

William Van Gaal, MD, Northern Hospital, Epping, Australia

Mathias-Christoph Brandt, MD, Uniklinikum Salzburg, Austria

Joëlle Kefer, MD, Cliniques Universitaires Saint-Luc (UCL), Belgium

Patrick Coussement, MD, AZ Sint-Jan Brugge, Belgium

Niels Thue Olsen, MD, Gentofte University Hospital, Denmark

Guillaume Cayla, MD, CHU de Nîmes, France

Etienne Puymirat, MD, Hôpital European Georges Pompidou, France, France

Gilles Lemesle, MD, CHRU de Lille, France

Didier Romain, MD, CHU de Brest, France

Michael Haude, MD, Lukaskrankenhaus Neuss, Germany

Thomas Schmitz, MD, Elisabeth-Krankenhaus Essen, Germany

Mohammad Sherif, MD, Universitätsklinikum der Charité, Campus Virchow-Klinikum, Germany

Jochen Wöhrle, MD, Klinikum Friedrichshafen GmbH, Germany

See Yue Arthur Yung, MD, Queen Mary Hospital, Hong Kong

Bryan Yan, MD, Prince of Wales Hospital, Hong Kong

András Vorobcsuk, MD, Moritz Kaposi General Hospital, Hungary

Ivan Horvath, MD, The University of Pécs, Hungary

Marco Ferlini, MD, Fondazione Irccs Policlinico San Matteo, Italy

Elisa Nicolini, MD, Azienda Ospedaliero Universitaria Ospedali Riuniti Umberto I - G.M. Lancisi, Italy

Andrejs Erglis, MD, Pauls Stradins Clinical University Hospital, Latvia

Artis Kalnins, MD, Riga East Clinical University Hospital, Latvia

Shaiful Azmi, MD, Institute Jantung Negara, Malaysia

Samer Somi, MD, HagaZiekenhuis, the Netherlands

Jithendra Somaratne, MD, Auckland City Hospital, New Zealand

Deanna Khoo Zhi Lin, MD, Tan Tock Seng Hospital – Cardiology, Singapore

Jose Maria de la Torre Hernandez, MD, Hospital Universitario Marques de Valdecilla, Spain

Alfonso Torres Bosco, MD, Hospital Clínico de Valencia, Spain

Juan Sanchis Fores, MD, Hospital Universitario de Araba, Spain

Stéphane Fournier, MD, Centre Hospitalier Universitaire Vaudoise, Switzerland

Juan Iglesias, MD, Hôpitaux Universitaires Genève (HUG), Switzerland

Vladimir Rubimbura, MD, Hôpital de Morges, Switzerland

Wirash Kehasukcharoen, MD, Central Chest Institute of Thailand, Thailand
